# Supervillin promotes epithelial-mesenchymal transition and metastasis of hepatocellular carcinoma in hypoxia via activation of the RhoA/ROCK-ERK/p38 pathway

**DOI:** 10.1186/s13046-018-0787-2

**Published:** 2018-06-28

**Authors:** Xueran Chen, Shangrong Zhang, Zhen Wang, Fengsong Wang, Xinwang Cao, Quan Wu, Chenggang Zhao, Huihui Ma, Fang Ye, Hongzhi Wang, Zhiyou Fang

**Affiliations:** 1 Anhui Province Key Laboratory of Medical Physics and Technology, Center of Medical Physics and Technology, Hefei Institutes of Physical Science, Chinese Academy of Sciences, No. 350, Shushan Hu Road, Hefei, 230031 Anhui China; 2 Hefei Cancer Hospital, Chinese Academy of Sciences, No. 350, Shushan Hu Road, Hefei, 230031 Anhui China; 3 University of Science and Technology of China, No. 96, Jin Zhai Road, Hefei, 230026 Anhui China; 4 School of Life Science, Anhui Medical University, No. 81, Mei Shan Road, Hefei, 230032 Anhui China; 5 Central Laboratory of Medical Research Center, Anhui Provincial Hospital, No. 17, Lu Jiang Road, Hefei, 230001 Anhui China; 6 Department of Radiation Oncology, First Affiliated Hospital, Anhui Medical University, No. 81, Mei Shan Road, Hefei, 230032 Anhui China

**Keywords:** Supervillin, RhoA/ROCK, ERK/P38, EMT, HCC

## Abstract

**Background:**

Hepatocellular carcinoma (HCC) is one of the most common malignant tumors in the world and metastasis is the leading cause of death associated with HCC. Hypoxia triggers the epithelial-mesenchymal transition (EMT) of cancer cells, which enhances their malignant character and elevates metastatic risk. Supervillin associates tightly with the membrane and cytoskeleton, promoting cell motility, invasiveness, and cell survival. However, the roles of supervillin in HCC metastasis remain unclear.

**Methods:**

Tissue microarray technology was used to immunohistochemically stain for supervillin antibody in 173 HCC tissue specimens and expression levels correlated with the clinicopathological variables. Tumor cell motility and invasiveness, as well as changes in the mRNA expression levels of genes associated with cancer cell EMT, were investigated. The relationship between supervillin and Rho GTPases was examined using Co-IP and GST pull-down.

**Results:**

Hypoxia-induced upregulation of supervillin promoted cancer cell migration and invasion via the activation of the ERK/p38 pathway downstream of RhoA/ROCK signaling. Furthermore, supervillin regulated the expression of EMT genes during hypoxia and accelerated the metastasis of HCC in vivo.

**Conclusions:**

Hypoxia-induced increase in supervillin expression is a significant and independent predictor of cancer metastasis, which leads to poor survival in HCC patients. Our results suggest that supervillin may be a candidate prognostic factor for HCC and a valuable target for therapy.

**Electronic supplementary material:**

The online version of this article (10.1186/s13046-018-0787-2) contains supplementary material, which is available to authorized users.

## Background

Hepatocellular carcinoma (HCC) is one of the most common malignancies, currently showing a poor prognosis [1–3]. Metastasis is the leading cause of death in majority of the HCC patients, of which the overall five-year survival rate is merely 5–6% [4–6]. The presence of a portal vein tumor thrombus (PVTT) is considered a strong predictor of metastasis and one of the most significant factors for a poor prognosis for HCC [7, 8]. In HCC, tumor cells rapidly grow and outpace their blood supply, leading to substantial hypoxia in the vicinity of HCCs, and this frequently accompanies PVTT [9]. Generally, hypoxia stabilizes hypoxia-inducible factors (HIFs), which are the master regulators of the cellular response to hypoxia through the regulation-associated gene transcription [10–12]. Hypoxic microenvironments have been known to induce the epithelial-mesenchymal transition (EMT) of cancer cells in various tumors [13, 14]. Tumor recurrence in HCCs is, in part, attributed to increased EMT, as well as enhanced tumor cell aggression and treatment resistance [15, 16]. Therefore, investigation of the mechanisms driving tumor EMT and cell migration/invasion is essential for the development of treatments for malignancies in HCC patients.

Several molecular players and pathways contribute to the modulation of the actin cytoskeleton, which affect EMT and metastasis during hypoxia [17]. Hypoxic microenvironments change the activity of Rho GTPases such as RhoA, Rac1, Cdc42, and downstream signaling molecules that regulate the dynamics of the actin cytoskeleton [18–20]. However, the effects of hypoxia on Rho GTPases and their activation vary greatly, which in turn control actin dynamics directly or by activating downstream signaling modules leading to activation of the RhoA/ROCK/cofilin or MLC/MLCK signal-transduction pathways [21–23]. ROCK could act as an upstream regulator activating the mitogen-activated protein kinase (MAPK) family, including p38, MAPK, and extracellular signal-regulated kinase (ERK) cascades [24–26]. Compromised MAPK signaling contributes to the pathology of many human diseases, including neurodegenerative disorders and cancer [27, 28]. The ERK or p38 signaling pathways play a key role in cancer development by stimulating cell proliferation and metastasis.

Supervillin is an actin and membrane-associated protein that has been implicated in each step of tumor cell migration and metastasis [29–31]. The supervillin family of proteins is the largest family in the villin/gelsolin superfamily. The supervillin proteins differ in their amino-termini as a result of alternative splicing of their pre-mRNAs, encoded by the *SVIL* gene. Cancer cells contain at least three supervillin isoforms (SV1, SV4, and SV5) [32–36]. These proteins are involved in actin filament assembly, cell spreading and lamellipodia extension, and focal adhesion maturation and/or disassembly [37–41]. Supervillin is also a component of invadosomes, where it regulates the invadosome half-life and matrix degradation, and enhances the secretion of matrix metalloproteinases (MMPs) [29–31]. Moreover, supervillin promotes cancer cell survival through integrin-based adhesions via its crosstalk between ERK-mediated survival signaling and cell motility pathways that contribute to ERK signaling [34, 35, 40]. Other mechanisms through which supervillin could promote cell motility, invasive activity, and cell survival remain unclear.

In this study, we explored the correlation between human HCC metastasis and the expression of supervillin in hypoxia. Here, we provide evidence that hypoxia-induced upregulation of supervillin promotes cancer cell migration and invasion while increasing the activation of RhoA. We also show that supervillin promotes ERK/p38 signal transduction as a downstream of the RhoA/ROCK signaling pathway, enhances the expression of EMT genes in HCC cells, and accelerates metastasis of HCC in vivo.

## Methods

### Antibodies

The primary antibodies described in this article include anti-supervillin (H340 [35]), anti-ERK1/2 (#4695; Cell Signaling Technology; MA, USA), anti-p-ERK1/2 (#4370; Cell Signaling Technology), anti-p38 (#8690; Cell Signaling Technology), anti-p-p38 (#4511; Cell Signaling Technology), anti-c-Jun N-terminal kinase (JNK)1/2 (#9252; Cell Signaling Technology), anti-p-JNK1/2 (#4668; Cell Signaling Technology), anti-E-cadherin (#sc7870; Santa Cruz Biotechnology, Inc.; CA, USA), anti-Vimentin (#sc73258; Santa Cruz Biotechnology, Inc.; CA, USA), anti-Snail1 (#sc393172; Santa Cruz Biotechnology, Inc), anti-β-actin (#3700; Cell Signaling Technology), and anti-β-tubulin (#TA506805; Origene; China).

### Immunohistochemical analyses of HCC tissue microarrays

HCC tissue microarrays were obtained from US Biomax, Inc. (Rockville, MD, USA). The immunohistochemical analyses of HCC tissue microarrays were carried out as previously described [42]. The KF-PRO Digital Slide Scanning System (Kongfong Biotech International Co., LTD; Ningbo, China) was used to visualize the signal.

### Cell culture, transfection, stable cell line, and treatment

HCC cell lines MHCC-97H, Huh-7, and HepG2 were a gift from Pro. Z.Y. Tang (Liver Cancer Institute, Fudan University, Shanghai) and were used in a previous study [42]. All the cell lines were kept at low passages for experimental use, and revived every 3 to 4 months. All cell lines used in this study were regularly authenticated by morphologic observation and tested for the absence of mycoplasma contamination. They were maintained in Dulbecco’s Modified Eagle’s Medium or DMEM (Hyclone; Logan, UT, USA) supplemented with 10% fetal bovine serum or FBS (Gibco; Grand Island, NY, USA) and 1% penicillin/streptomycin (Hyclone). Cells were exposed to hypoxia (1.0% O_2_) in a hypoxic chamber (Thermo Fisher Scientific, Inc., Waltham, MA, USA) for the indicated period.

Cells were transfected with supervillin Stealth siRNA #1, #2, #3, and #4 and negative control siRNA (Invitrogen; Carlsbad, CA, USA) at 40 nM using Lipofectamine® RNAiMAX (Invitrogen), or with GFP-tagged SV1, SV4, and SV5 plasmids with the BTX ECM® 830 Electroporation System (Harvard Apparatus; Holliston, MA, USA), according to the manufacturer’s instructions. The RNAi targeting sequences and their corresponding target exons in the supervillin gene are shown in Additional file 1: Table S1.

MHCC-97H supervillin knockdown stable clones were constructed using pLVshRNA-EGFP (2A) Puro (Invivogen, Shanghai, China) lentiviral shRNA vector targeting supervillin (5′-AGGTGATGAAGCCAGATGA-3′).

To determine the functional crosstalk between RhoA/ROCK and ERK/p38 signaling, HCC cells were pretreated with the specific mitogen-activated protein kinase kinase (MEK) inhibitor (PD0325901, 10 μM; MedChemExpress, Monmouth Junction, NJ, USA), p38 MAPK inhibitor (SB239063, 10 μM; MedChemExpress), or ROCK inhibitor (Y27632 2HCl, 10 μM; MedChemExpress) for 1 h. To investigate the functional crosstalk between supervillin expression and HIF1α, we used the HIF inhibitor (2-Methoxyestradiol; 2-MeOE2, 10 μM; MedChemExpress) for 8 h.

### Hepatic artery ligation (HAL) and orthotopic liver implantation

A 100-μl cell suspension (MHCC-97/SVIL-shRNA, containing 5 × 10^6^ cells) in PBS was subcutaneously injected into the flanks of male BALB/c nude mice (*n* = 5) at the age of 6 weeks. Mice were sacrificed 2 weeks later and the tumors were cut into approximately 1 mm^3^ for successive orthotopic implantation into 6-week-old male BALB/c nude mice. To restrict blood flow into the liver and mimic hypoxia, a HAL was carried out by tying a fine thread around the main branch of the hepatic artery. For tumors that were formed from GFP-labeled cell lines, the growth of the tumor was monitored by bioluminescence detection using IVIS 100 Imaging System (Xenogen; Princeton, NJ, USA). Six weeks after the orthotopic implantation, mice were sacrificed, and their lungs and livers were excised. The fluorescence intensity from tumor foci was taken as the tumor metastasis potential for comparison among transplanted nude mice. Animal experiments were performed according to the guidelines of the Animal Use and Care Committees at Hefei Institutes of Physical Science, CAS.

### Migration and invasion assays

Transwell chambers with 8-μm pores (Corning; NY, USA) were used for cell migration assay. For the tumor cell invasion assay, the Transwell membrane was pre-coated with 30 μl of Matrigel matrix (1:3 mixed with PBS; BD Biosciences; Heidelberg, Germany). Cell suspension in serum-free medium was added to the upper chamber, and then incubated in normoxia or hypoxia for 18 h. After incubation, migrated or invaded cells were fixed with methanol for 30 min, stained with 0.1% crystal violet, and counted under a light microscope at 100× magnification (Leica, DMI 4000 B; Germany). Then, the crystal violet was eluted by methanol and the OD at 546 nm was examined using a spectrophotometer.

### Immunoprecipitation

Cell lysate preparation and immunoprecipitation was conducted as previously described [43], using 30 μl Anti-HA Magnetic Beads (Pierce Biotechnology, Rockford, IL, USA). Twenty microliters of beads were resuspended in 20 μl of 2× protein sample buffer before analysis.

### Immunofluorescence

Cells were washed, fixed on glass slides, and stained with primary antibodies: anti-E-cadherin (1:200), anti-Vimentin (1:250), and anti-Snail1 (1:100). The secondary antibodies conjugated with fluorescein (FITC; Invitrogen; Carlsbad, CA, USA) were then incubated for 1 h at room temperature and stained with DAPI. Stained cells were then visualized under a fluorescence microscope (Leica, DMI 4000 B; Germany).

### RhoA, Cdc42, and Rac1 activation assay

GTP loading assays were carried out as described [41]. Briefly, cells were extracted in lysis buffer at 4 °C and centrifuged for 10 min at 14000 g. Supernatants were incubated with GST-RBD or GST-PBD pre-bound to glutathione-Sepharose beads for 1 h at 4 °C. GST-peptide beads were centrifuged, washed thrice in washing buffer A (25 mM Tris, 40 mM NaCl, 30 mM MgCl_2_, 10 μg/ml leupeptin, 10 μg/ml aprotinin, 1 mM 4-(2-aminoethyl)-benzene sulfonyl fluoride, pH 7.5), boiled in sample buffer, and resolved by SDS-PAGE.

### Statistical analysis

Statistical calculations were performed using Prism 5 software (GraphPad). Data were expressed as mean ± SD, unless otherwise indicated. Continuous variables were evaluated using an unpaired Student’s t-test for comparisons between two groups. The Pearson’s chi-square test was used to analyze the association of supervillin expression with clinicopathological parameters. Multivariate analysis was performed using forward stepwise logistic regression analysis. Two-sided tests were performed with a *p* < 0.05 indicating statistical significance: ns, non significant, * - p < 0.05, ** - *p* < 0.01, *** - *p* < 0.001.

## Results

### Supervillin is positively associated with human HCC metastasis

**Fig. 1:**
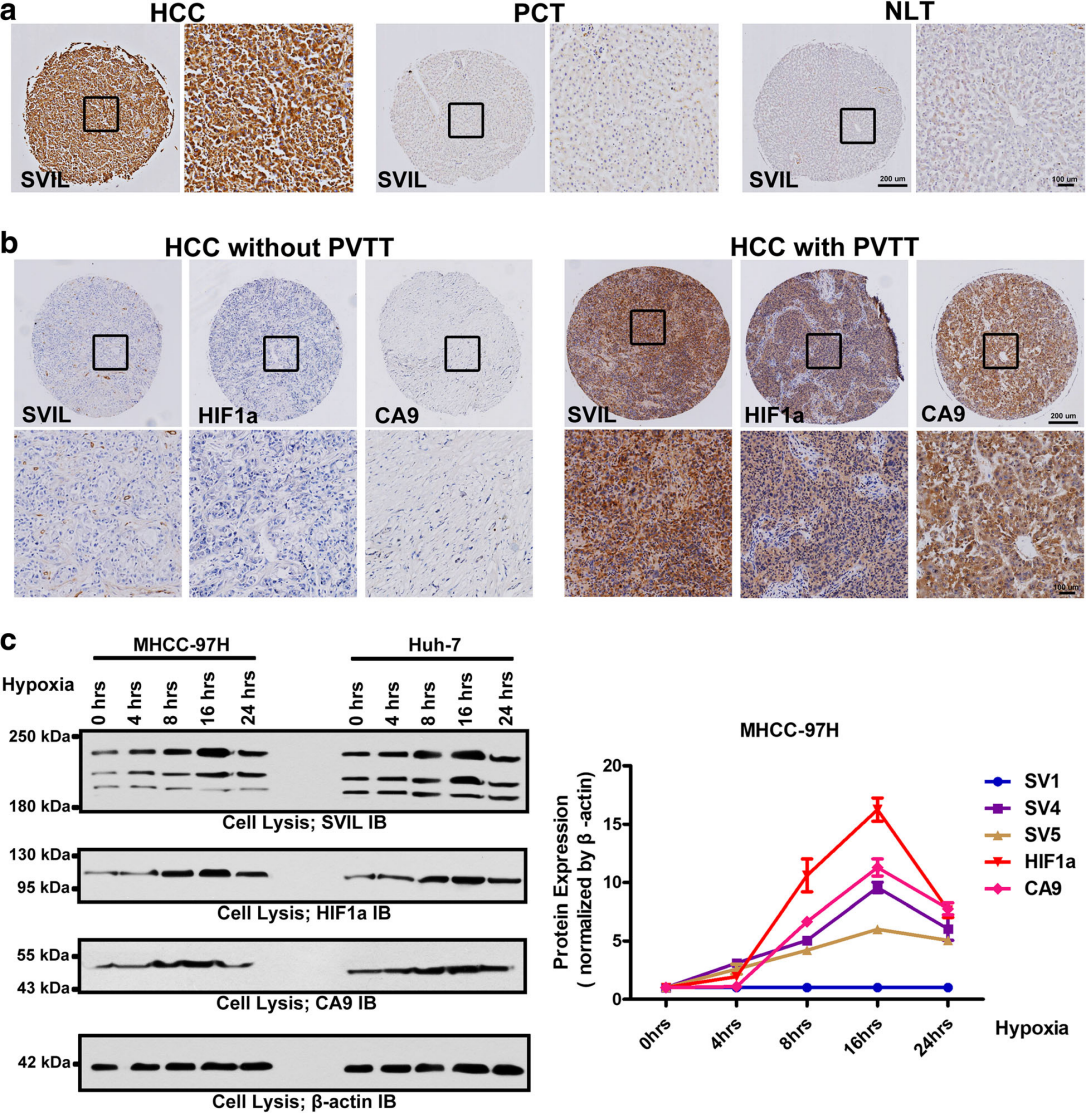
Supervillin is positively associated with portal vein tumor thrombus (PVTT) development and metastasis in hepatocellular carcinoma (HCC) patients. **a**. Immunohistochemical (IHC) staining showing upregulation of supervillin in HCC, compared with para-carcinoma tissue (PCT), and normal liver tissue (NLT). The left image is under 40× magnification and the right image (inset box) is under 100× magnification (black). **b**. IHC staining showing the accumulation of HIF1α, CA9 and supervillin in a tumor with PVTT, compared with a tumor without PVTT. The upper image is under 40× magnification and the lower image (inset box) is under 100× magnification (black). **c**. Western blot analysis showing the expression of supervillin, HIF1α and CA9 in MHCC-97H and Huh-7 cells. β-actin was used as the loading control. Data represent the mean of at least three independent experiments ± SD in MHCC-97H cells

To investigate the prognostic value of supervillin expression levels in HCCs, we performed microarray-based immunohistochemical analyses of 173 HCC tissues from two independent cohorts with comparable clinicopathological features and complete follow-up data. In 124 HCC specimens, supervillin expression levels were dramatically upregulated, compared to those in adjacent non-tumor specimens or normal liver tissues (Fig. 1a). Notably, a higher expression of supervillin was significantly correlated with the presence of PVTT (χ2 = 15.44, p < 0.01), occurrence of serosal infiltration (χ2 = 12.64, p < 0.01), and distant metastasis (χ2 = 9.79, p < 0.01), which indicated the potential important roles of supervillin played in HCC metastasis (Table 1).

**Table 1:** Clinicopathologic correlation of supervillin expression in human HCC

Clinical Pathological Parameters	n	SVIL	Χ^2^	P
Gender				
Male	132	94(71.2%)	0.06	>0.05
Female	41	30(73.2%)		
Age (Years)				
≥ 45	118	83(70.3%)	0.33	>0.05
<45	55	41(74.5%)		
AFP (ng/L)				
≤ 200	23	13(56.5%)	3.11	>0.05
>200	57	20(35.1%)		
Tumor Size (cm)				
≤ 3	24	12(50.0%)	1.14	>0.05
>3~ 10	41	15(36.6%)		
>10	15	6(40.0%)		
Grade of Differentiation				
Well-differentiated	18	13(72.2%)	0.07	>0.05
Moderately-differentiated	110	77(70.0%)		
Poorly-differentiated	39	28(71.2%)		
Portal Vein Tumor Thrombus				
Yes	33	30(91.0%)	15.44	<0.01
NO	40	19(47.5%)		
Serosal Infiltration				
Yes	35	28(80.0%)	12.64	<0.01
NO	30	11(36.7%)		
Distant Metastasis				
Yes	29	23(80.6%)	9.79	<0.01
NO	20	7(35.0%)		

### Hypoxia-induced upregulation of supervillin promotes cell migration and invasiveness of HCC

PVTT is a significant risk factor for metastasis in HCC patients and frequently accompanies substantial hypoxia within the tumor microenvironment [44]. Supervillin was positively expressed in hypoxic PVTT (Fig. 1b). Thus, we first examined the expression profiles of supervillin during hypoxia. We quantified the levels of supervillin splicing variants, the hypoxia-response factor, HIF1α, and the downstream target of HIF1α, CA9, in lysates from Huh-7 (low metastatic HCC cell line) and MHCC-97H (high metastatic HCC cell line) cells as a function of exposure time to a reduced (1%) oxygen environment (Fig. 1c). As expected, HIF1α and CA9 proteins gradually accumulated due to a hypoxia-induced increase in protein stability. Strikingly, the levels of the supervillin isoforms SV4 and SV5 reached a peak after 16 h (SV4, *p* < 0.001; SV5, *p* < 0.001) and then gradually declined during hypoxia, as did HIF1α. By contrast, the level of SV1 did not change significantly during hypoxia. Our previous study showed that a reduction in SV4 and SV5 levels decreased the rates of cell migration and invasion of human A549 lung carcinoma cells during normoxia [36]. This suggests that increased SV4 and SV5 expression during hypoxia may play a role in the metastatic potential of HCC.

**Fig. 2:**
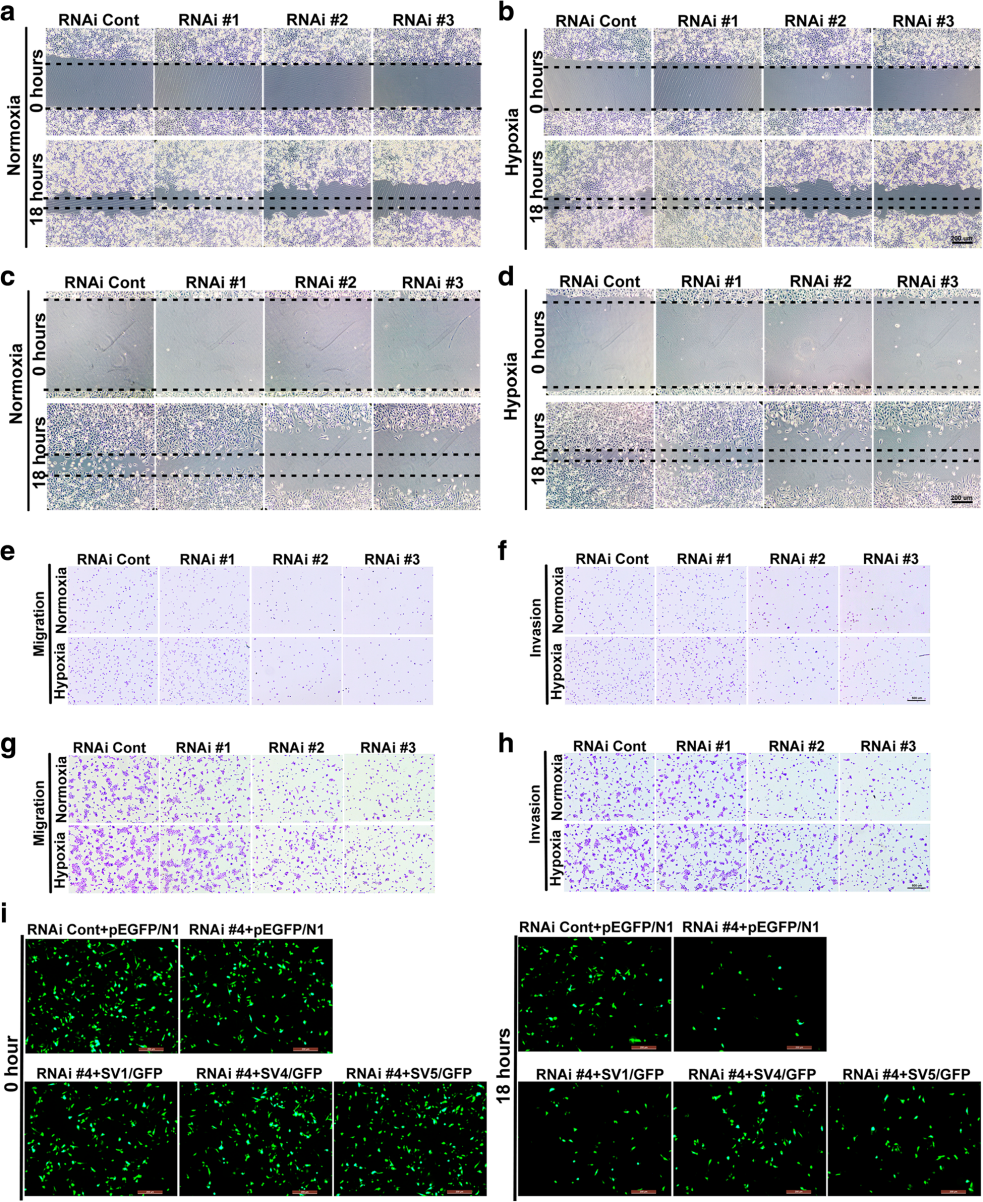
Supervillin promotes HCC migration and invasion during hypoxia. **a-d.** Huh-7 (**a**, **b**) and MHCC-97H (**c**, **d**) cell mobility was detected by wound healing assay. Cells were transfected with control or supervillin-specific siRNA and incubated under normoxic conditions for 48 h, scratched and exposed to normoxia, or hypoxia for 18 h, respectively. The closure of the scratch was monitored and photographed. Scale bar = 200 μm. **E, G.** Huh-7 (**e**) and MHCC-97H (**g**) cell migration were detected by Boyden Chamber Transwell assays. Cells were transfected with control or supervillin-specific siRNA and incubated under normoxic conditions for 48 h, after which they were seeded into Transwell chambers for 18 h under normoxia or hypoxia. The number of migrated cells was monitored and photographed. **f, h.** Huh-7 (F) and MHCC-97H (H) cell invasion were detected by Boyden Chamber Transwell assays. Cells were transfected with control or supervillin-specific siRNA and incubated under normoxic conditions for 48 h, seeded into Matrigel-coated Transwell inserts, and incubated under normoxia or hypoxia for 18 h. The number of invaded cells was monitored and photographed. **i.** MHCC-97H cells that had been treated with supervillin-specific siRNA (RNAi #4, targeted for the supervillin 3′UTR) were allowed to recover their expression of SV1, SV4, and SV5 before assay for cell migration in Transwell chambers under hypoxia for 18 h. The migrated cells on the upper side (at 0 h) and lower side (18 h) were monitored and photographed

To determine the potential role of supervillin, especially the different supervillin splicing isoforms on HCC cell migration and invasion under hypoxic conditions, we used Stealth RNAi™ dsRNAs that target sequences within *SVIL* coding exon 4 (RNAi #1; specific for SV4), coding exon 5 (RNAi #2; targets both SV4 and SV5), coding exon 10 (RNAi #3; targets all three isoforms), and the 3’-UTR (RNAi #4; targets all three isoforms). As described previously [36], each Stealth siRNA that targeted supervillin splice isoforms (SV1, SV4, and SV5) in HCC cells reduced the level of each isoform by ≥75% (Additional file 1: Figure S1A-C). Indeed, hypoxia caused 17 and 41% increases in the rates of cell migration of Huh-7 cells (Fig. 2a and b, and Additional file 1: Figure S2A, 282 ± 4.42 μm in hypoxia vs. 208 ± 2.85 μm in normoxia; *p* < 0.01) and MHCC-97H cells (Fig. 2c and d, and Additional file 1: Figure S2B, 431 ± 6.15 μm in hypoxia vs. 382 ± 4.15 μm in normoxia; *p* < 0.01), respectively. Transfection of supervillin-specific RNAi #2 or #3 resulted in an 81 and 83% reduction in cell migration in Huh-7 and MHCC-97H cells after 18 h in normoxia, respectively (Fig. 2a and c, and Additional file 1: Figure S2A and B). In RNAi #2 or #3-transfected cells, cell migration increased by only 23 and 22% after 18 h of hypoxia, respectively (Fig. 2b and d, and Additional file 1: Figure S2A and B). These results suggest that a reduction in the levels of supervillin isoforms, especially SV4 and SV5, blocks hypoxia-induced increases in cell migration rates, as well as reducing cell migration in normoxia.

To strengthen our findings from the wound-healing model, we further studied cell migration and invasion using the Boyden chamber method. The results showed that cell migration increased by 36 and 86% (Fig. 2e and g, and Additional file 1: Figure S2C and D) and cell invasion by 58 and 26% (Fig. 2f and h, and Additional file 1: Figure S2E and F) in Huh-7 and MHCC-97H cells under hypoxic stress, respectively. Supervillin knockdown with RNAi #2 or #3 resulted in a 65 and 72% reduction in cell migration (Fig. 2e and g, and Additional file 1: Figure S2C and D) and a 73 and 59% reduction in cell invasion (Fig. 2f and h, and Additional file 1: Figure S2E and F) for Huh-7 and MHCC-97H cells after 18 h in normoxia, respectively, and almost completely abolished hypoxia-induced increases in HCC cell migration and invasion. Consistent with these findings, overexpression of SV1, SV4, or SV5 in supervillin knockdown cells rescued cell migration rates to different degrees (Fig. 2i, and Additional file 1: Figure S2G, 21% increase after SV1 overexpression; *p* < 0.01, 43% increase after SV4 overexpression; *p* < 0.01, and 39% increase after SV5 overexpression; *p* < 0.01). These results support the involvement of supervillin during the migration and matrix invasion of HCC cells under hypoxic stress.

### Supervillin is essential for the hypoxia-induced EMT in HCC cell lines

**Fig. 3:**
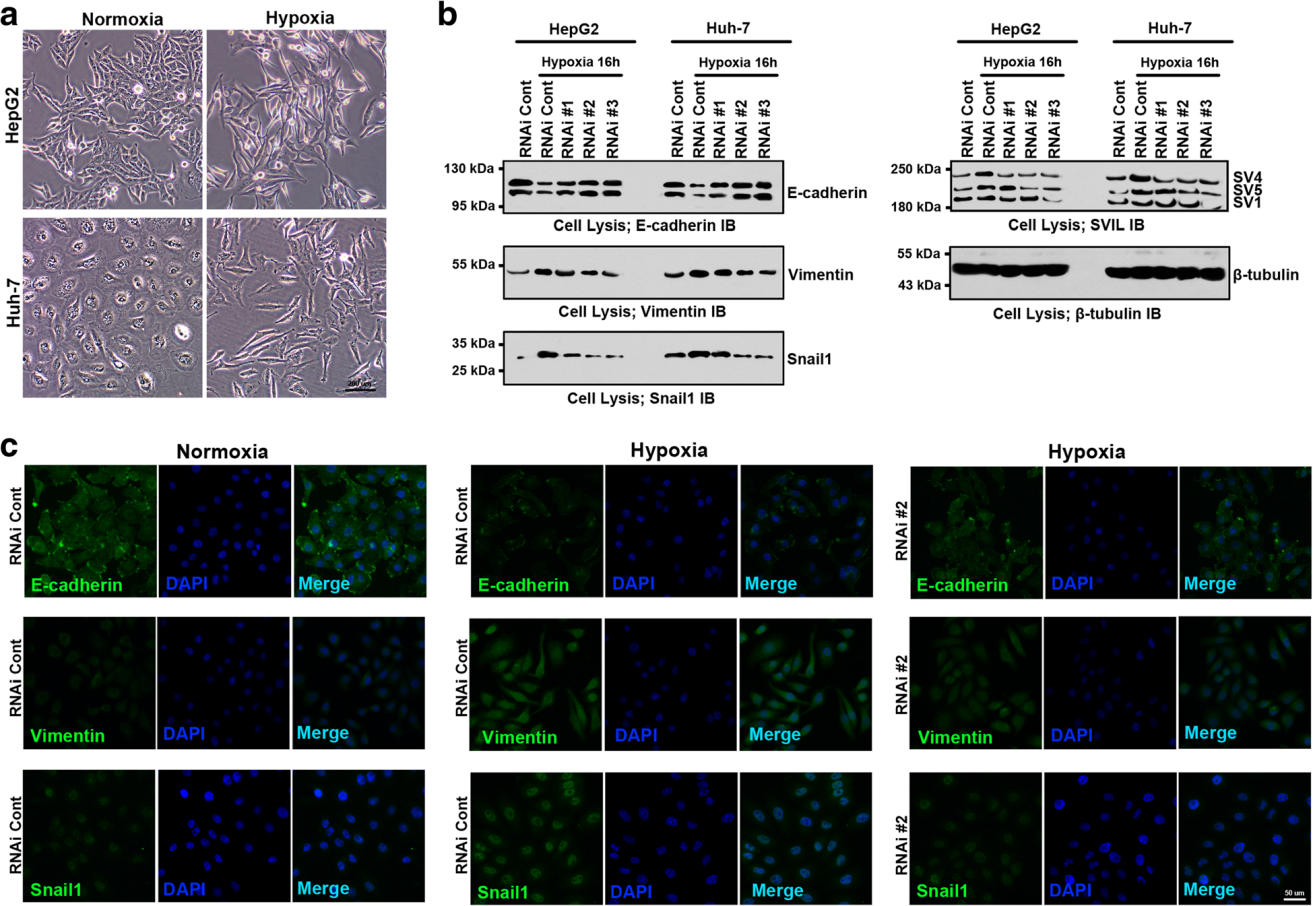
Supervillin regulates hypoxia-induced epithelial-mesenchymal transition (EMT) of HCC. **a.** Bright-field microscopy showing the morphological changes that occur when HepG2 and Huh-7 cells are cultured for 16 h in hypoxia. **b.** Immunoblots showing the changes in E-cadherin, Vimentin, Snail1, and supervillin isoforms during hypoxia after treatment with supervillin-specific siRNA. HepG2 and Huh-7 cells were transfected with a control or supervillin-specific siRNA and incubated under normoxia for 48 h followed by being scratched and exposed to normoxia or hypoxia for 16 h; β-tubulin was used as the loading control. **c.** Immunofluorescence staining showing that the relative amounts and localization of E-cadherin, Snail1, and Vimentin under normal and hypoxic conditions in Huh-7 cells treated with control or supervillin-specific siRNA. Scale bar = 50 μm

Given that the EMT program is critical for matrix invasion and the dissemination of most and possibly all carcinoma types, we examined whether supervillin expression induces EMT in hypoxic HCC cell lines. After exposure to 1% oxygen for 16 h, Huh-7 and HepG2 cells became elongated (Fig. 3a). Expression of the epithelial biomarker E-cadherin was significantly down-regulated, with simultaneous rapid increase in the expression of the mesenchymal biomarkers Vimentin and Snail1 in both Huh-7 and MHCC-97H cells (Fig. 3b and Additional file 1: Figure S3A-C). During hypoxia, the amount of E-cadherin and its localization at the plasma membrane were reduced, whereas the signals for Vimentin and Snail1 became stronger than those in normoxia (Fig. 3c and Additional file 1: Figure S3D). These results are consistent with hypoxia-stimulated EMT in HCC cells [44, 45]. RNAi-mediated down-regulation of supervillin in Huh-7 and MHCC-97H cells largely prevented the hypoxia-induced decrease in E-cadherin levels and the increase in Vimentin and Snail1 signaling (Fig. 3b and c, and Additional file 1: Figure S3A-F).

During epithelial to mesenchymal transition (EMT), cancer cells gain migratory and invasive properties due to the dramatic reorganization of the actin cytoskeleton [10–12]. As shown in Additional file 1: Figure S3G-H, there was a significant increase in F-actin during hypoxia, as compared with that in normoxia. However, the reduction of supervillin decreased the number and thickness of stress fibers during hypoxia (Additional file 1: Figure S3I). These results suggest that supervillin plays an important role in the hypoxia-induced EMT in HCC, and is responsible for the rearrangement of actin cytoskeleton under hypoxia.

### Supervillin-mediated HCC cell migration and invasion involves the RhoA/ROCK pathway during hypoxia

**Fig. 4:**
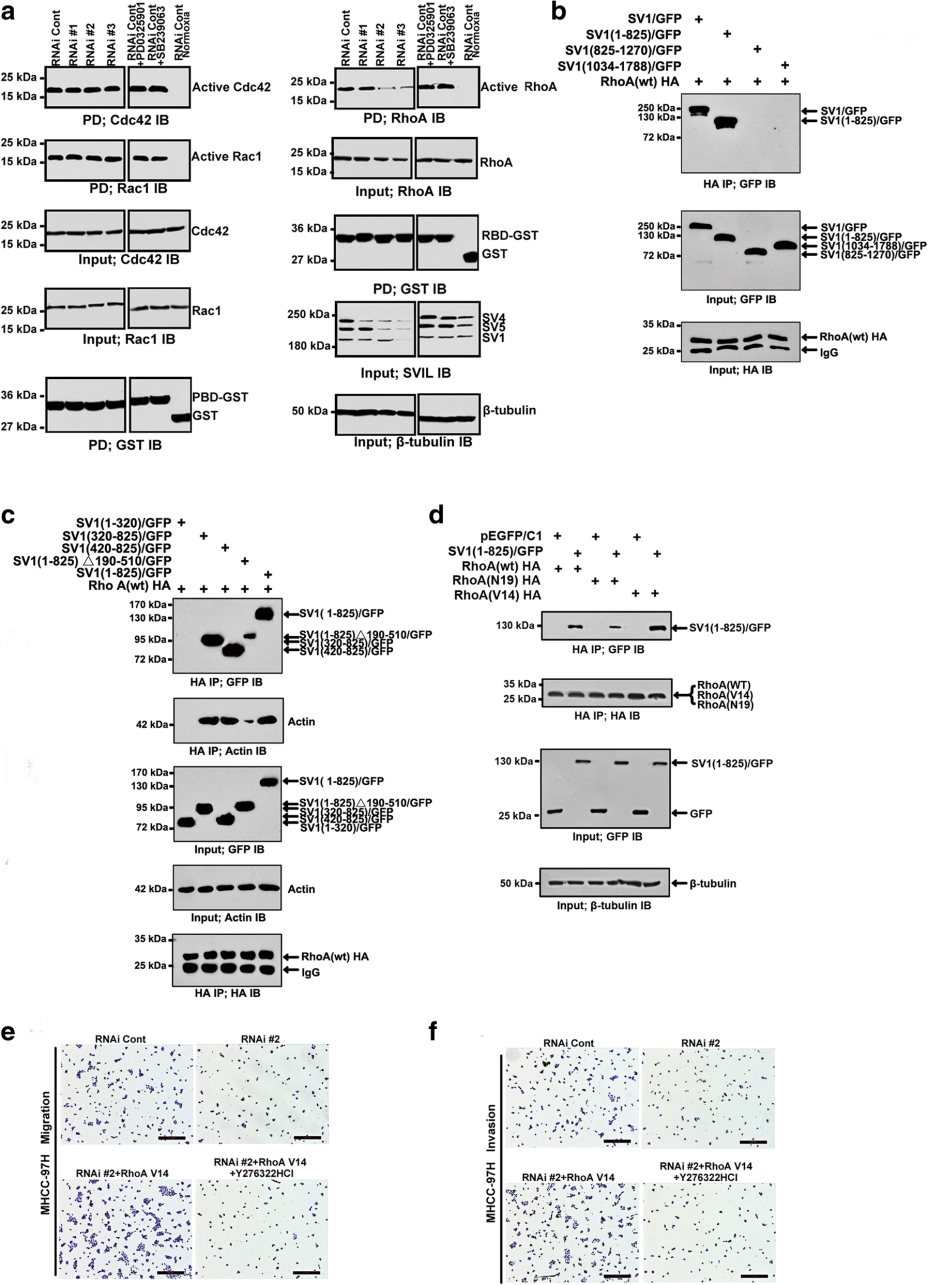
Supervillin-mediated HCC cell migration and invasion involves the RhoA/ROCK pathway during hypoxia. **a.** MHCC-97H cells were transfected with control or supervillin-specific siRNA for 48 h, exposed to hypoxia for 16 h, and lysates were assayed for the relative amounts of GTP-loaded (activated) Rac1, Cdc42, and RhoA. Cells that had been transfected with control RNAi were treated with a MEK inhibitor (PD0325901) or a p38 inhibitor (SB239063) before assaying for GTP-Rac1, Cdc42, and RhoA levels (right). **b.** The interaction between supervillin and RhoA. Cell lysates were prepared from HEK293 cells co-transfected with GFP-tagged supervillin and HA-tagged RhoA, as described in the Methods. Immunoprecipitation (IP) and immunoblotting (IB) were performed with anti-HA or anti-GFP antibodies. **c.** Mapping of the binding region of supervillin and RhoA. HEK293 cells were transfected with GFP-tagged supervillin and HA-tagged RhoA. Twenty-four hours after transfection, cells were harvested and lysed, and RhoA was immunoprecipitated with anti-HA. Pellets and lysates were immunoblotted with anti-HA or anti-GFP antibodies. **d.** The interaction between supervillin and RhoA. Total HEK293 cell lysates containing GFP-tagged SV1 (1–825) and HA-tagged RhoA(WT), RhoA(V14), or RhoA(N19) were prepared. IP and IB were performed with anti-HA or anti-GFP antibodies. **e.** MHCC-97H cells that had been transfected with control or supervillin-specific RNAi were treated with the ROCK inhibitor Y27632 2HCl, incubated for 18 h in 1% O_2_, and tested for migratory activity in Boyden Chamber Transwell assays. **f.** MHCC-97H cells that had been transfected with control or supervillin-specific siRNA were treated with the ROCK inhibitor Y27632 2HCl, exposed to hypoxia for 18 h, and tested for cell invasion through Matrigel in Boyden Chamber Transwell assays

During hypoxia, the small G proteins of the Rho family are involved in the reorganization of the actin cytoskeleton and cell migration [18, 46]. To explore the mechanism of supervillin action during HCC migration and invasion, we first examined the crosstalk with Rho GTPases known to regulate cell-substrate adhesion, cell polarization, and the rates of cell spreading and translocation. We screened for functional crosstalk between supervillin and Rac1, Cdc42, and RhoA (Fig. 4a, and Additional file 1: Figure S4A and B). As has been described previously in HeLa cervical carcinoma cells, supervillin knockdown with RNAi #2 or #3 decreased Rac1 loading in normoxia [41]. During hypoxia, knockdown of supervillin with RNAi #2 or RNAi #3 was accompanied by a significant reduction in the amount of GTP-loaded (active) RhoA, as assessed by pulldown assays with GST-tagged Rho-binding domain (RBD). In contrast, no significant changes were observed in GTP loading of Cdc42. When supervillin expression was allowed to recover to initial levels, GTP-RhoA levels almost returned to control values (Additional file 1: Figure S5A). Next, we identified if RhoA-GTP is a novel direct or indirect interaction partner of supervillin (Fig. 4b and Additional file 1: Figure S5B). Notably, the N-terminus of supervillin co-precipitated with RhoA, especially the region containing the three documented F-actin binding domains [37]. Supervillin preferentially bound to active RhoA (WT and the constitutively active V14 mutant; Fig. 4c and d), suggesting that this association might play an important role during cytoskeletal rearrangements in hypoxia. Consistent with this hypothesis, expression of the RhoA(V14) mutant reversed the inhibitory effects of supervillin knockdown on cell migration (Fig. 4e, and Additional file 1: Figure S4C and D, *p* < 0.01 in Huh-7 and MHCC-97H cells) and invasion (Fig. 4f, and Additional file 1: Figure S4C and D, p < 0.01 in Huh-7 and MHCC-97H cells) during hypoxia. When HCC cells were pretreated with the ROCK inhibitor (Y27632 2HCl), RhoA(V14)-induced rescues of cell migration and invasion were blocked (Fig. 4e and f, and Additional file 1: Figure S4c and d, *p* < 0.01 in Huh-7 and MHCC-97H cells), implying that supervillin-induced increase in HCC cell migration and invasion during hypoxia require RhoA/ROCK signal transduction.

### ERK/p38 acts downstream of RhoA/ROCK in supervillin-induced cell migration and invasion

**Fig. 5:**
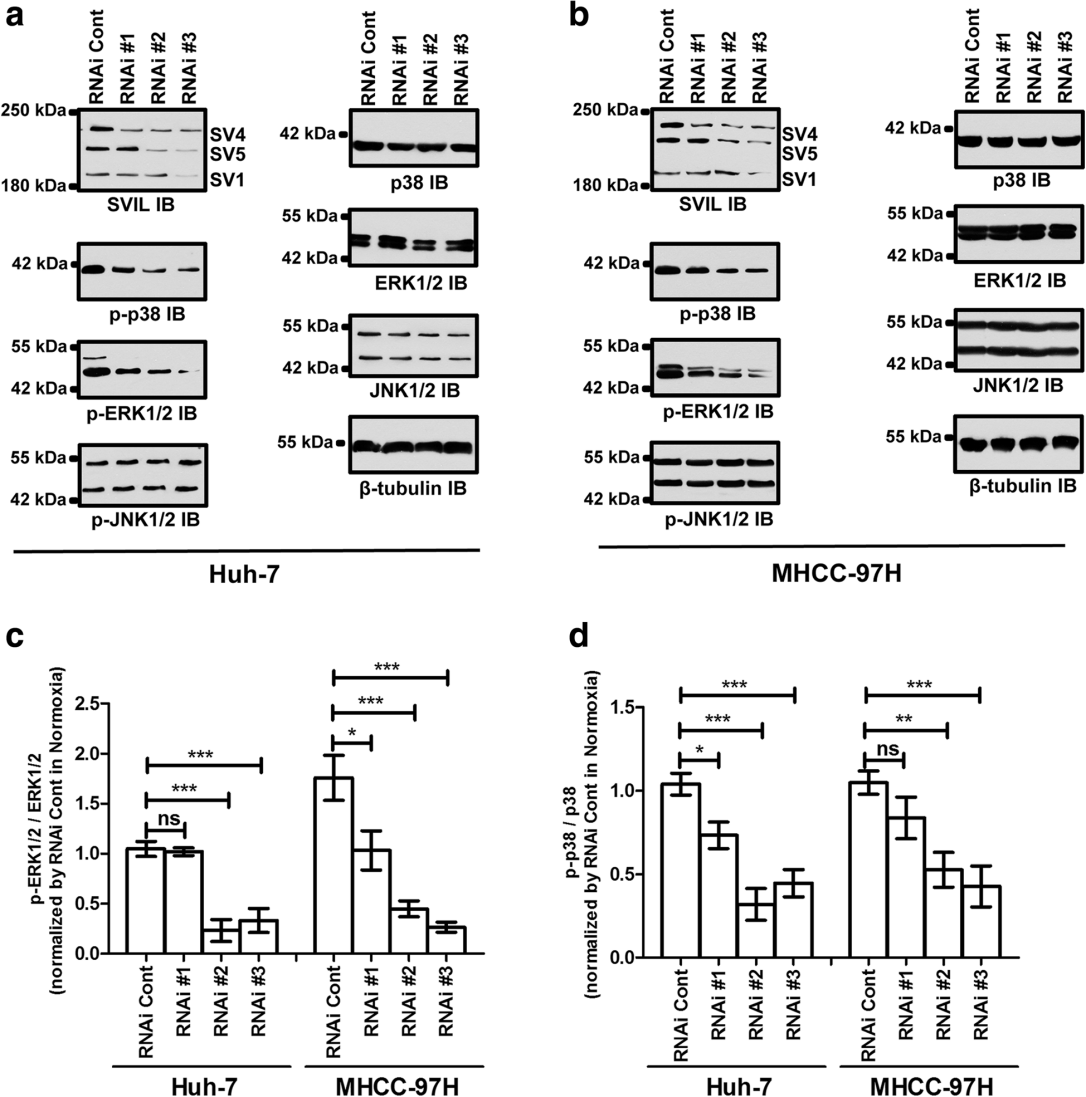
Supervillin activates ERK/p38 signaling in normoxia and hypoxia. **a, b.** Huh-7 (**a**) and MHCC-97H (**b**) cells were transfected with control or supervillin-specific siRNA and exposed to hypoxia for 16 h, and then the changes in phosphorylated p38, ERK, and JNK were detected by western blot. β-tubulin was used as the loading control. **c.** Quantification of the p-ERK1/2 level in hypoxic Huh-7 and MHCC-97H cells transfected with control or supervillin-specific siRNA. Each p-ERK1/2 level was normalized to the level in hypoxic cells transfected with control siRNA. **d.** Quantification of the p-p38 level in hypoxic Huh-7 and MHCC-97H cells transfected with control or supervillin-specific siRNA. Each p-p38 level was normalized to the level in hypoxic cells transfected with control siRNA. Data represent the mean of at least three independent experiments ± SD

**Fig. 6:**
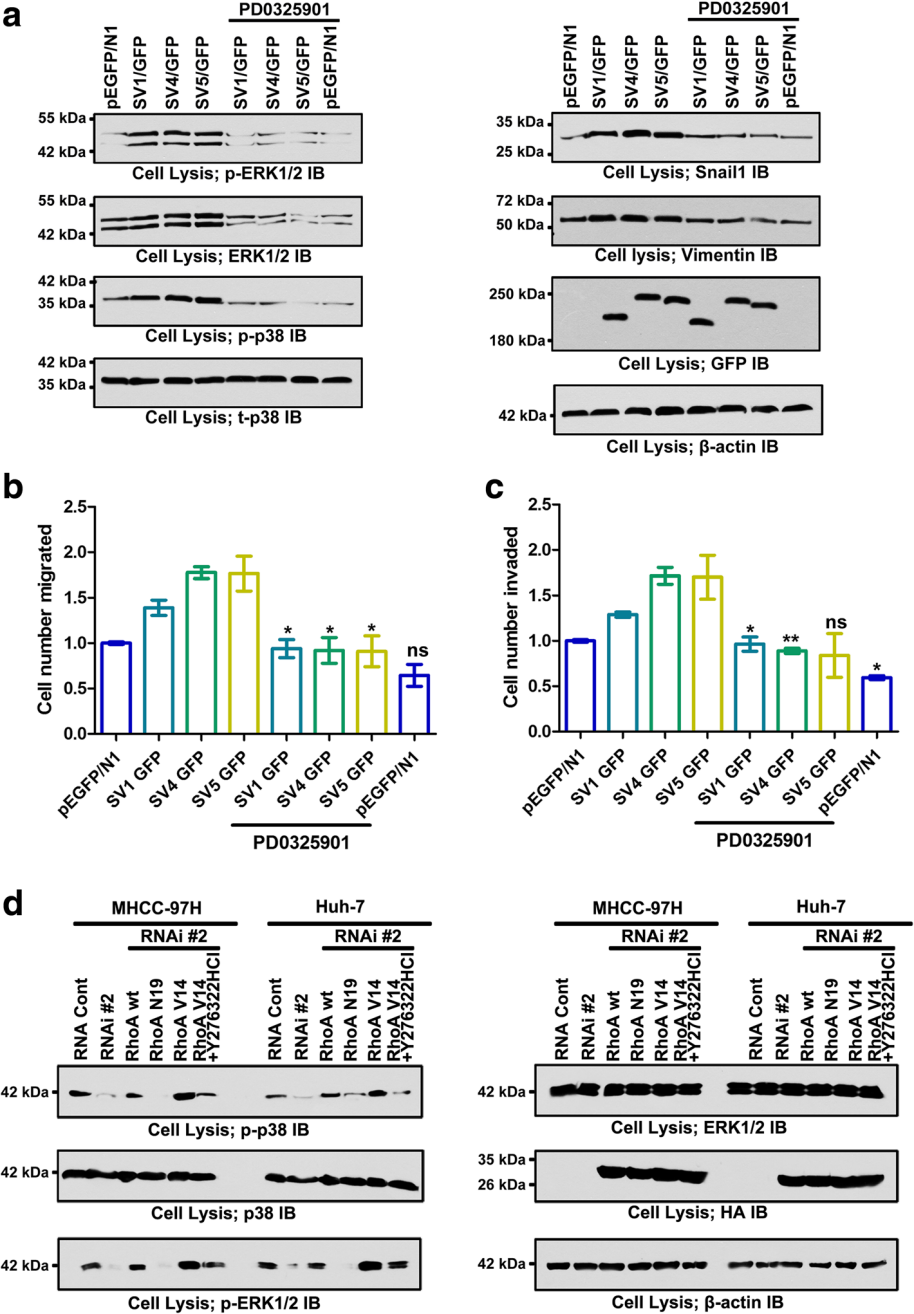
ERK/p38 is downstream of RhoA/ROCK in supervillin-mediated cell metastasis during hypoxia. **a.** MHCC-97H cells that had been transfected with SV1, SV4, or SV5 plasmids were treated with PBS or the MEK inhibitor PD0325901 (10 μM) for 1 h under hypoxic conditions before assaying for phosphorylated p38, phosphorylated ERK, Snail1, and Vimentin by immunoblotting. β-tubulin was used as the loading control. **b, c.** MHCC-97H cells that had been transfected with SV1, SV4, or SV5 plasmids were treated with PBS or the MEK inhibitor PD0325901 (10 μM) for 1 h under hypoxic conditions before assay for cell migration (**b**) and invasion (**c**). The number of migrated SV1, SV4, or SV5 cells treated with PD0325901 was compared to those control cells treated with PBS. **D.** MHCC-97H and Huh-7 cells co-transfected with control or supervillin-specific siRNA and a RhoA(WT), RhoA(V14), or RhoA(N19) plasmids for 48 h were treated with PBS or the ROCK inhibitor Y27632 2HCl (10 μM) for 16 h during hypoxia, and then assayed for phosphorylated p38 and ERK by immunoblotting. β-actin was used as the loading control

In migrating cells, supervillin and associated proteins coordinate a rapid, basolateral membrane recycling pathway that contributes to ERK signaling and actin-based cell motility [35]; the supervillin isoform SV3 has been proposed to scaffold ERK with its upstream kinase MEK in smooth muscle cells [47]. To further elucidate supervillin-associated mechanisms in hypoxia, we monitored changes in the MAPK/ERK1/2 and associated signal molecules in Huh-7 and MHCC-97H HCC cells (Fig. 5). Inhibition of supervillin expression induced a decrease in phosphorylated p38 and ERK1/2 to different degrees under hypoxic stress. Expression of supervillin isoforms rescued these decreases after supervillin knockdown in Huh-7 cells (Additional file 1: Figure S5C). In addition, overexpression of individual supervillin isoforms during hypoxia increased the levels of both total and phosphorylated ERK1/2 (Fig. 6a). The increases in both total and phosphorylated ERK1/2 were reversed with the MEK inhibitor PD0325901 (Fig. 6a). In addition, phosphorylation of p38, Vimentin, and Snail1 were also inhibited by PD0325901 (Fig. 6a). HCC cell migration and invasion ability was blocked after the addition of 10 μM PD0325901 for 18 h during hypoxia (Fig. 6b and c). These data show that the MAPK/ERK/p38 signaling cascade participates in the supervillin-driven HCC cell migration and invasion within the hypoxic microenvironment.

Both RhoA/ROCK and ERK/p38 signaling are indispensable for cell migration and invasion; therefore, we investigated potential crosstalk between the RhoA/ROCK and ERK/p38 signaling pathways. We found that phosphorylation of ERK and p38 depended on the RhoA/ROCK pathway because the levels of ERK/p38 activation were affected by the presence of active RhoA and various inhibitors (Fig. 6d and Additional file 1: Figure S6). The expression of active RhoA(V14) increased ERK/p38 phosphorylation relative to control values, even in the presence of RNAi #2. The ROCK inhibitor (Y27632 2HCl) abolished the RhoA-induced increase in ERK and p38 phosphorylation in MHCC-97H and Huh-7 cells under hypoxic conditions. However, the MEK inhibitor (PD0325901) and p38 inhibitor (SB239063) had no effect on RhoA/ROCK activation (Fig. 4a, and Additional file 1: Figure S4A and B), indicating that ERK/p38 phosphorylation is downstream of RhoA/ROCK activation. Collectively, our results suggest that supervillin promotes cell mobility through the RhoA/ROCK and ERK/p38 pathways within hypoxic microenvironments.

### Expression of supervillin in HCC enhances tumor metastasis in vivo

**Fig. 7:**
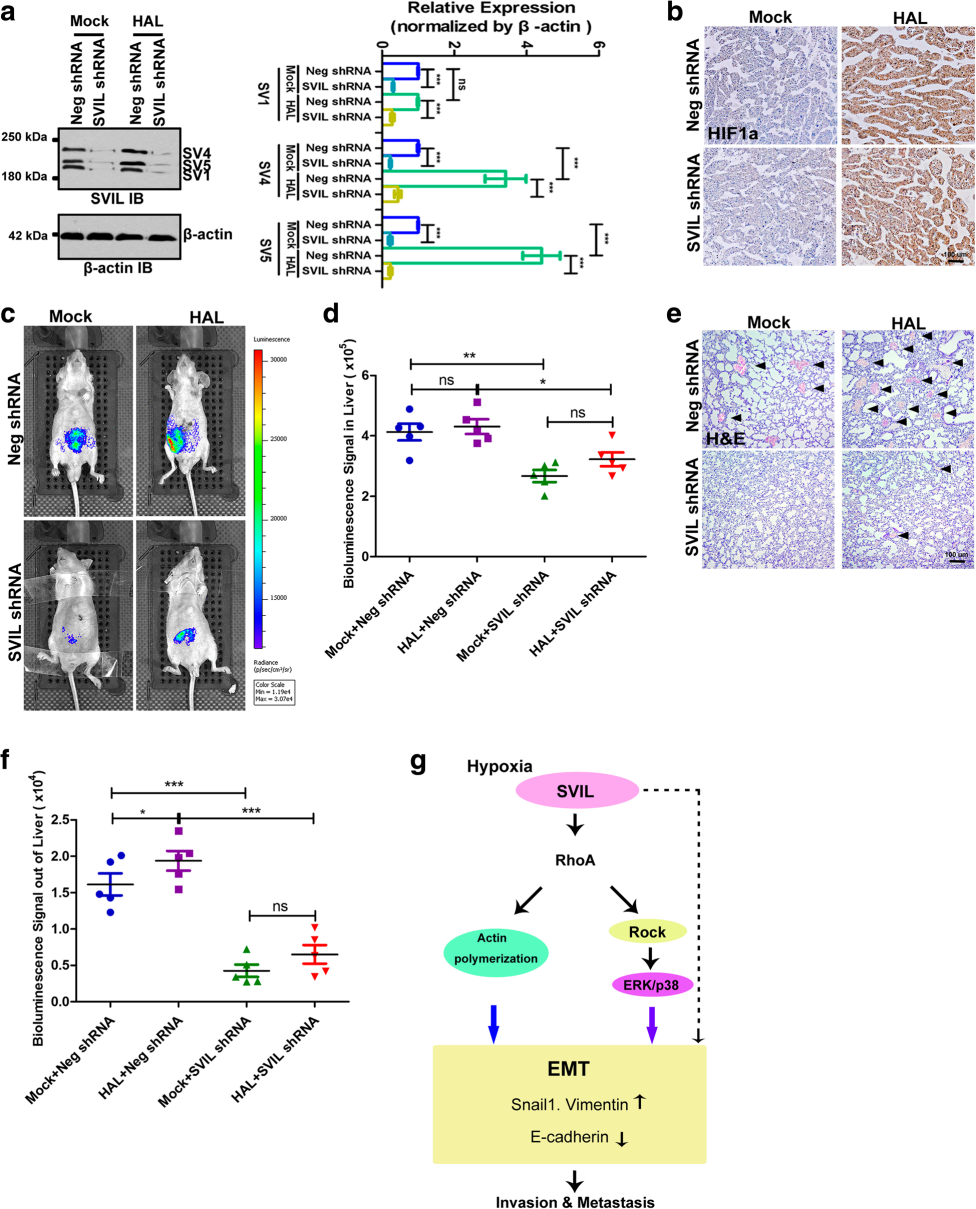
Knockdown of supervillin inhibits HCC metastasis. Mice (*n* = 5) were implanted with a ~ 1 mm^3^ tumor “seed” derived from a supervillin-knockdown clone or a clone treated with a control shRNA; mice were treated with or without hepatic artery ligation (HAL). **a** After 6 weeks, each tumor was dissected, lysed, and the level of supervillin was determined by immunoblotting. β-actin was used as the loading control. Data represent the mean of at least three independent experiments ± SD in MHCC-97H cells. **b** IHC staining showing the accumulation of HIF1α in mice with HAL, compared with those without HAL. Scale bar = 100 μm. **c** Bioluminescence imaging of representative mice at the end of the experiment. **d** Bioluminescence signal intensity in dissected livers was compared among the four experimental groups. **e** Hematoxylin/eosin staining of lung tissues. Arrows indicate the metastatic tumor foci in the lung tissues. Scale bar = 100 μm. **f** The bioluminescent signal intensities in the lungs were compared among the four experimental groups. **g** A schematic diagram showing the signaling axis of supervillin-RhoA/ROCK-ERK/p38 in HCC. Under hypoxia, supervillin levels increase and lead to RhoA activation. Activated RhoA regulates actin polymerization, induces the ROCK-ERK/p38 signaling pathway, and EMT, thereby promoting HCC cell migration, invasiveness, and metastasis

To explore a potential role for supervillin in HCC metastasis in vivo, we carried out orthotopic liver implantation experiments. Tumor seeds derived from MHCC-97H with non-target control or supervillin-knockdown cells were implanted into the livers of mice, after either HAL or a mock surgery (Mock). HAL increased the stability of the HIF1α protein and the amount of supervillin, especially in the SV4 and SV5 isoforms (Fig. 7a and b), similar to our results using a hypoxic incubator (Fig. 1d). HAL treatment enhanced tumor growth and lung metastasis in vivo (Fig. 7c-f). The largest number of metastatic tumor foci was observed in the lungs of animals subjected to HAL (Fig. 7e and f). The metastatic capability was suppressed in mice implanted with supervillin-knockdown cells (Fig. 7e and f). The suppressed metastatic capability was consistent with a decrease in phosphorylated p38 and ERK1/2 to different degrees under hypoxic stress (Additional file 1: Figure S7). Indeed, knockdown of supervillin was accompanied by a significant reduction in the amount of GTP-loaded (active) RhoA (Additional file 1: Figure S7). These results suggest that supervillin contributes significantly to cell motility and metastasis of HCC cells under hypoxic stress in vivo, as well as in vitro.

## Discussion

Prevention and effective control of metastasis are essential to HCC therapy; thus, there is an urgency to improve our understanding of the molecular mechanisms of HCC progression and metastasis. Supervillin is an actin-associated protein that regulates the actin dynamics by interacting with myosin II, F-actin, and cortactin to promote cell contractility and cell motility [29, 39, 48]. However, the roles of supervillin in HCC metastasis remain unclear. The results of the current study demonstrated that hypoxia elicits an upregulation in the expression of supervillin, which was a significant and independent predictor of cancer metastasis and poor survival in HCC patients. Moreover, the RhoA/ROCK-ERK/p38 signaling pathway and RhoA-mediated actin polymerization are systematically connected, contributing to HCC cell metastasis mediated by supervillin (Fig. 7g).

We show here that supervillin is up-regulated in HCC cells during hypoxia and PVTT. Supervillin expression is dramatically increased in HCC tumor specimens, compared to adjacent non-tumor specimens or normal liver tissues. Notably, the higher expression of supervillin was positively correlated with the presence of PVTT, which suggested that supervillin production might be induced by the hypoxic tumor microenvironment. Hypoxia triggers the stability of HIF-1α, which has previously been shown to induce EMT in cancer cells and promote metastasis [13, 14]. Indeed, the expression of supervillin isoforms, especially SV4 and SV5, increased significantly in HCC cells under hypoxic conditions in vitro and in vivo. The up-regulation of SV4 and SV5 is not dependent on HIF-mediated transcriptional activation (Additional file 1: Figure S1D and E), which suggests that supervillin promotes the EMT of HCC cells through an alternative pathway. Supervillin knockdown blocked EMT in HCC cells and decreased the rates of HCC cell migration and invasion during hypoxia. In a mouse model, HAL treatment enhanced total tumor growth and lung metastasis with control, but not with supervillin knockdown, HCC cells. These data are consistent with previous studies, in which supervillin was shown to dynamically regulate focal adhesion turnover, podosome/invadopodia structure reorganization, and matrix degradation [29, 31, 37]. Our results suggest that supervillin contributes significantly to cell motility and metastasis of HCC cells both in vitro and in vivo under hypoxic stress.

Actin-associated proteins are intricately involved in the organization and assembly of the actin cytoskeleton and thus regulate the cellular processes of adhesion, migration, and invasion [49, 50]. These cellular processes are commonly dysregulated during cancer development and progression to drive tumor metastasis and invasion. Since this organization of actin structures is mediated by Rho proteins such as Cdc42, Rac1, and RhoA, we decided to investigate the relationship between Rho GTPases and supervillin function in HCC cell lines under hypoxic conditions. Our results suggest that, during hypoxia, supervillin forms a complex with activated RhoA and supervillin may enhance RhoA activation, or stabilize activated RhoA. The overexpression of constitutively active RhoA(V14) mostly rescues the inhibitory effects of supervillin knockdown on cell migration and invasion during hypoxia. RhoA(V14) overexpression-mediated increases in cell motility were inhibited by the ROCK inhibitor Y27632 2HCl, providing evidence that supervillin promotes HCC cell migration and invasion via the RhoA/ROCK signaling cascade. In fibroblasts, ROCK inhibition blocked MLC phosphorylation, and resulted in immature focal adhesions at the periphery and migrates less effectively [51]. These results are also consistent with the demonstrations that supervillin overexpression disrupts stress fiber formation [37, 38], which could alter RhoA localization within the cells and supervillin-mediated effects on cytoskeleton rearrangements and mechanical cell contractility via RhoA/ROCK/MLCK signal transduction pathways [29, 39].

As reported, ROCK acts as an upstream regulator leading to the activation of MAPKs, including p38, MAPK, and ERK [25, 27, 28]. Thus, we analyzed the main molecules involved in the MAPK/ERK/p38 pathway and cancer metastasis, and our evidence shows that supervillin promotes the activation of ERK1/2 and p38, but not of JNK1/2 in hypoxic HCC cells. Overexpression of supervillin proteins SV1, SV4, or SV5 can rescue the activation of ERK1/2 and p38, which further affects the expression of EMT-related genes such as Snail1 and Vimentin in HCC cells during hypoxia, and enhances the cell migration and invasion. Pretreatment with the MEK inhibitor PD0325901 reversed the supervillin-induced activation of ERK1/2 and p38, and the inhibition of cell migration or invasion. Moreover, active RhoA(V14) significantly increased the activation of ERK1/2 and p38 in supervillin knockdown HCC cells, but this effect was abolished by the ROCK inhibitor Y27632 2HCl. However, overexpression of active Rac1(G12) suppressed the activation of ERK/p38 (Additional file 1: Figure S8), which suggested RhoA and Rac1 might antagonistically contribute to EMT and HCC cell metastasis [52]. Additionally, p38 activation under hypoxia might be responsible for the up-regulation of select MMPs in HCCs. In pancreatic cancer cells, the p38 MAPK pathway regulates MMP-7 activity, which is in turn responsible for the enhanced cancer cell motility and invasion [53]. Also, MMP12 is regulated by the p38 MAPK pathway in chondrosarcoma cells [54]. These data establish one possible mechanism by which supervillin promotes the metastasis of HCC, and that is through MAPK/ERK/p38 activation via the downstream RhoA-ROCK signaling pathway.

## Conclusion

To our knowledge, our study is the first to demonstrate that supervillin is an independent prognosis indicator for HCC patients. In addition, in vitro and in vivo assays verified the essential role of supervillin in promoting invasion, metastasis, and EMT by activating the RhoA/ROCK-ERK/p38 signaling pathway during hypoxia. Indeed, the suppression of supervillin expression had an effect similar to that of ROCK or MAPK inhibition. Therefore, our study suggests that supervillin can be used as a valuable prognostic biomarker and a potential therapeutic target in HCC.

## Additional file

Additional file 1: **Table S1.** Stealth siRNA used in the study. **Figure S1.** siRNA that targeted supervillin splice isoforms (SV1, SV4, and SV5) reduced the level of each isoform in HCC cells, and the up-regulation of supervillin was not dependent on HIF1α-induced transcriptional activation. **Figure S2.** Supervillin promotes HCC migration and invasion during hypoxia. **Figure S3.** Quantification of EMT markers’ expression levels regulated by supervillin during hypoxia. **Figure S4.** Supervillin regulated HCC cell migration and invasion through the RhoA/ROCK pathway during hypoxia. **Figure S5.** Supervillin regulates RhoA activation and ERK/p38 transduction during hypoxia. **Figure S6.** Quantification of phosphorylated p38 and ERK levels regulated by supervillin-RhoA/ROCK signaling during hypoxia. **Figure S7.** Supervillin-RhoA/ROCK-ERK/p38 signaling pathway in HCC metastasis under hypoxia *in vivo*. **Figure S8.** Supervillin promotes Rac1 activity and Rac-mediated cell metastasis in normoxia and hypoxia. **Figure S9.** RhoA activation and MAPK signaling vary in cell types and environments. **Figure S10.** There was not obvious effect on microtubule dynamics upon supervillin knockdown. **Figure S11.** The inhibitors used in this study did not affect cell apoptosis and viability. (PDF 2231 kb)

## Abbreviations

EMT: epithelial-mesenchymal transition
ERK: extracellular signal-regulated kinase
JNK: c-Jun NH2-terminal kinase
RhoA: Ras homolog gene family, member A
ROCK: Rho-associated, coiled-coil containing protein kinase
RT-PCR: Reverse transcription polymerase chain reaction
SV1: Supervillin isoform 1
SV4: Supervillin isoform 4
SV5: Supervillin isoform 5
UTR: Untranslated region
