# Correction: Supervillin promotes epithelialmesenchymal transition and metastasis of hepatocellular carcinoma in hypoxia via activation of the RhoA/ROCK-ERK/p38pathway

**DOI:** 10.1186/s13046-026-03820-5

**Published:** 2026-09-05

**Authors:** Xueran Chen, Shangrong Zhang, Zhen Wang, Fengsong Wang, Xinwang Cao, Quan Wu, Chenggang Zhao, Huihui Ma, Fang Ye, Hongzhi Wang, Zhiyou Fang

**Affiliations:** 1 Anhui Province Key Laboratory of Medical Physics and Technology, Center of Medical Physics and Technology, Hefei Institutes of Physical Science, Chinese Academy of Sciences, No. 350, Shushan Hu Road, Hefei, 230031 Anhui China; 2 Hefei Cancer Hospital, Chinese Academy of Sciences, No. 350, Shushan Hu Road, Hefei, 230031 Anhui China; 3 University of Science and Technology of China, No. 96, Jin Zhai Road, Hefei, 230026 Anhui China; 4 School of Life Science, Anhui Medical University, No. 81, Mei Shan Road, Hefei, 230032 Anhui China; 5 Central Laboratory of Medical Research Center, Anhui Provincial Hospital, No. 17, Lu Jiang Road, Hefei, 230001 Anhui China; 6 Department of Radiation Oncology, First Affiliated Hospital, Anhui Medical University, No. 81, Mei Shan Road, Hefei, 230032 Anhui China

**Correction: J Exp Clin Cancer Res 37, 128 (2018)**

**https://doi.org/10.1186/s13046-018-0787-2**

Following publication of the original article [1], authors identified an error in Fig. 2i (0 h, RNAi#4 + SV5/GFP), where an image from a different experimental condition was inadvertently used. The correct image has been substituted in the revised figure. This correction does not affect the interpretation or conclusions of the study.

**Incorrect** Fig. 2i

**Fig. 2:**
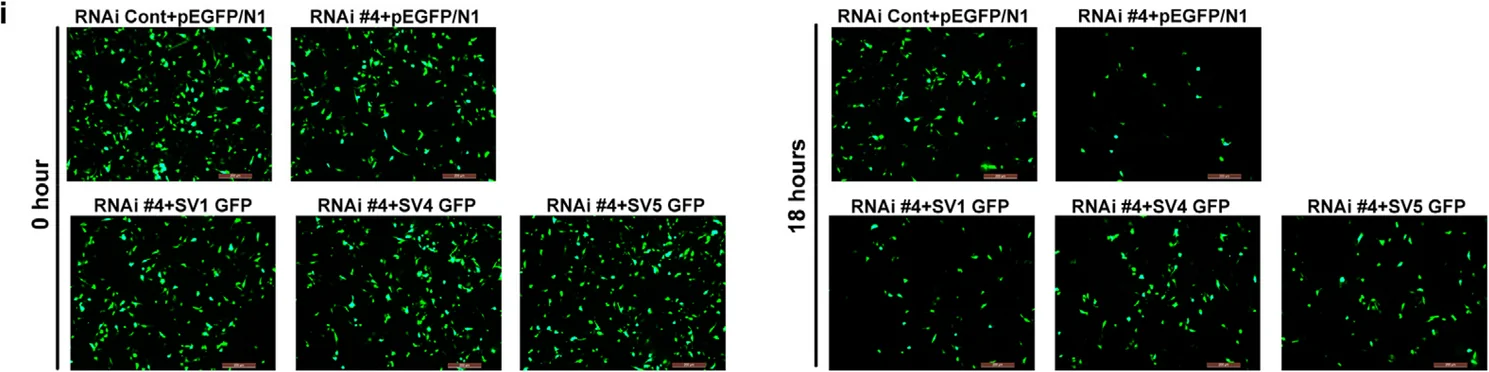
Supervillin promotes HCC migration and invasion during hypoxia. **a**-**d**. Huh-7 (a, b) and MHCC-97H (**c**, **d**) cell mobility was detected by wound healing assay. Cells were transfected with control or supervillin-specific siRNA and incubated under normoxic conditions for 48 h, scratched and exposed to normoxia, or hypoxia for 18 h, respectively. The closure of the scratch was monitored and photographed. Scale bar = 200 μm. **E**, **G**. Huh-7 (**e**) and MHCC-97H (**g**) cell migration were detected by Boyden Chamber Transwell assays. Cells were transfected with control or supervillin-specific siRNA and incubated under normoxic conditions for 48 h, after which they were seeded into Transwell chambers for 18 h under normoxia or hypoxia. The number of migrated cells was monitored and photographed. **f**, **h**. Huh-7 (F) and MHCC-97H (H) cell invasion were detected by Boyden Chamber Transwell assays. Cells were transfected with control or supervillin-specific siRNA and incubatedunder normoxic conditions for 48 h, seeded into Matrigel-coated Transwell inserts, and incubated under normoxia or hypoxia for 18 h. The number of invaded cells was monitored and photographed. **i**. MHCC-97H cells that had been treated with supervillin-specific siRNA (RNAi #4, targeted for the supervillin 3′UTR) were allowed to recover their expression of SV1, SV4, and SV5 before assay for cell migration in Transwell chambers under hypoxia for 18 h. The migrated cells on the upper side (at 0 h) and lower side (18 h) were monitored and photographed

**Correct** Fig. 2i

**Fig. 2:**
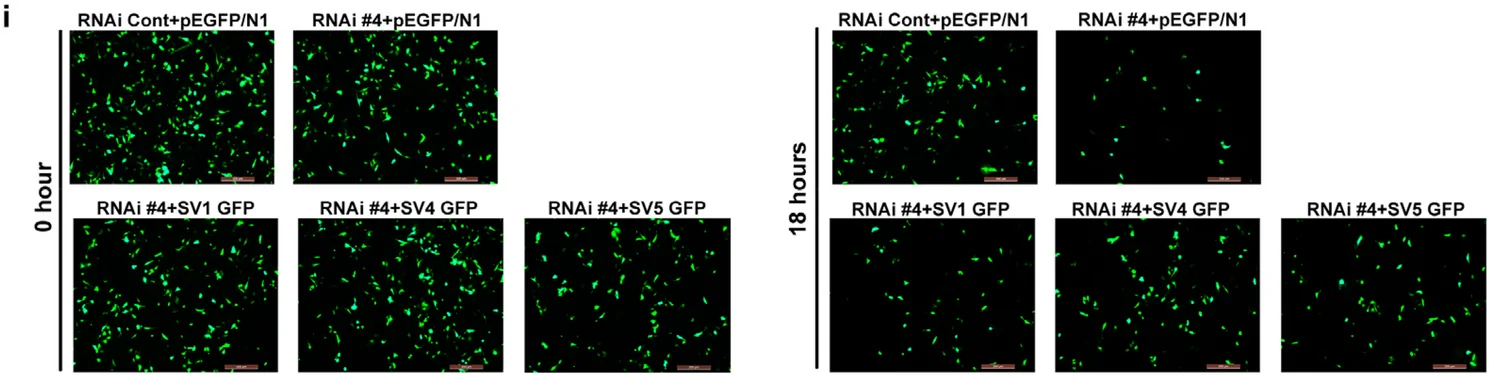
Supervillin promotes HCC migration and invasion during hypoxia. **a**-**d**. Huh-7 (a, b) and MHCC-97H (**c**, **d**) cell mobility was detected by wound healing assay. Cells were transfected with control or supervillin-specific siRNA and incubated under normoxic conditions for 48 h, scratched and exposed to normoxia, or hypoxia for 18 h, respectively. The closure of the scratch was monitored and photographed. Scale bar = 200 μm. **E**, **G**. Huh-7 (**e**) and MHCC-97H (**g**) cell migration were detected by Boyden Chamber Transwell assays. Cells were transfected with control or supervillin-specific siRNA and incubated under normoxic conditions for 48 h, after which they were seeded into Transwell chambers for 18 h under normoxia or hypoxia. The number of migrated cells was monitored and photographed. **f**, **h**. Huh-7 (F) and MHCC-97H (H) cell invasion were detected by Boyden Chamber Transwell assays. Cells were transfected with control or supervillin-specific siRNA and incubatedunder normoxic conditions for 48 h, seeded into Matrigel-coated Transwell inserts, and incubated under normoxia or hypoxia for 18 h. The number of invaded cells was monitored and photographed. **i**. MHCC-97H cells that had been treated with supervillin-specific siRNA (RNAi #4, targeted for the supervillin 3′UTR) were allowed to recover their expression of SV1, SV4, and SV5 before assay for cell migration in Transwell chambers under hypoxia for 18 h. The migrated cells on the upper side (at 0 h) and lower side (18 h) were monitored and photographed
