# Supplementary material for: Supervillin promotes epithelial-mesenchymal transition and metastasis of hepatocellular carcinoma in hypoxia via activation of the RhoA/ROCK-ERK/p38 pathway
Source: J Exp Clin Cancer Res. 2018 Jun 28;37:128. doi: 10.1186/s13046-018-0787-2 (PMC6025706; doi:10.1186/s13046-018-0787-2)
Supplement: Supplementary file 1 — Table S1. Stealth siRNA used in the study. Figure S1. siRNA that targeted supervillin splice isoforms (SV1, SV4, and SV5) reduced the level of each isoform in HCC cells, and the up-regulation of supervillin was not dependent on HIF1α-induced transcriptional activation. Figure S2. Supervillin promotes HCC migration and invasion during hypoxia. Figure S3. Quantification of EMT markers’ expression levels regulated by supervillin during hypoxia. Figure S4. Supervillin regulated HCC cell migration and invasion through the RhoA/ROCK pathway during hypoxia. Figure S5. Supervillin regulates RhoA activation and ERK/p38 transduction during hypoxia. Figure S6. Quantification of phosphorylated p38 and ERK levels regulated by supervillin-RhoA/ROCK signaling during hypoxia. Figure S7. Supervillin-RhoA/ROCK-ERK/p38 signaling pathway in HCC metastasis under hypoxia in vivo. Figure S8. Supervillin promotes Rac1 activity and Rac-mediated cell metastasis in normoxia and hypoxia. Figure S9. RhoA activation and MAPK signaling vary in cell types and environments. Figure S10. There was not obvious effect on microtubule dynamics upon supervillin knockdown. Figure S11. The inhibitors used in this study did not affect cell apoptosis and viability. (PDF 2231 kb) [file 13046_2018_787_MOESM1_ESM.pdf]

Supplementary Figure S1

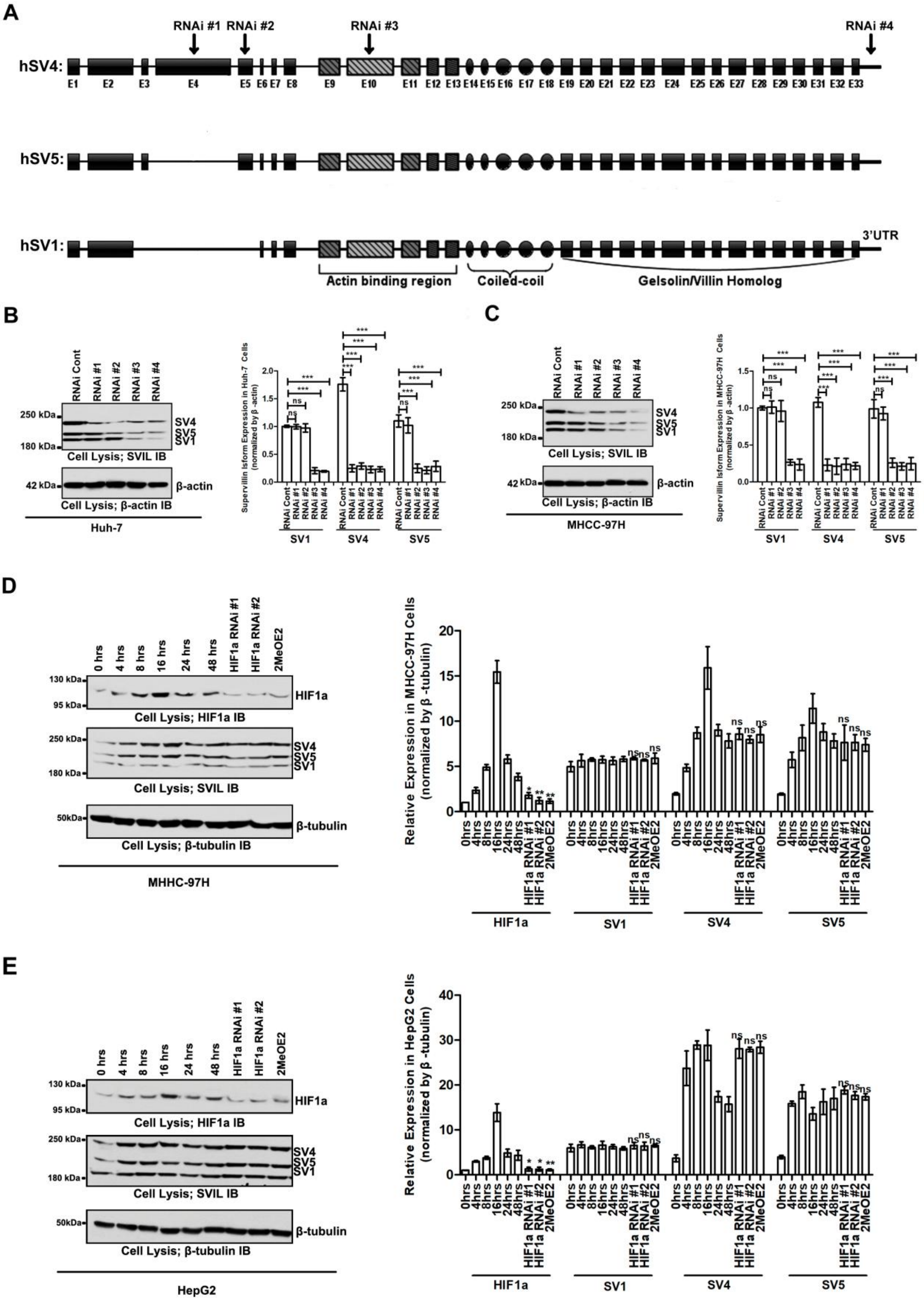

Supplementary Figure S1. siRNA that targeted supervillin splice isoforms (SV1, SV4, and SV5) reduced the level of each isoform in HCC cells, and the up-regulation of supervillin was not dependent on HIF1α-induced transcriptional activation. A. A diagram of supervillin splicing isoforms (coding

exons) and the locations of the sites targeted by the siRNA are indicated on the diagram. **B, C.** Huh-7 (B) and MHCC-97H (C) cells were transfected with control or supervillin-specific RNAi for 48 h, and then lysates were assayed for the expression of supervillin using western blot analysis. β-actin was used as the loading control. Quantification of SV1, SV4, or SV5 level in Huh-7 and MHCC-97H cells transfected with control or supervillin-specific RNAi. Each supervillin splice isoform level was normalized to the level of β-actin. Data represent the mean of at least three independent experiments ± SD in Huh-7 and MHCC-97H cells. **D, E.** MHHC-97H (D) and HepG2 (E) cells were transfected with HIF1a-specific RNAi or treated with HIF inhibitor 2MeOE under 8-hour hypoxia, and then lysates were assayed for the expression of HIF1a and supervillin using western blot analysis. β-tubulin was used as the loading control. Quantification of HIF1a, SV1, SV4, or SV5 level in MHCC-97H and HepG2 cells transfected with HIF1a-specific RNAi or treated with HIF inhibitor 2MeOE. Each supervillin splice isoform level was normalized to the level of β-tubulin. Data represent the mean of at least three independent experiments ± SD in MHCC-97H and HepG2 cells.

Supplementary Figure S2

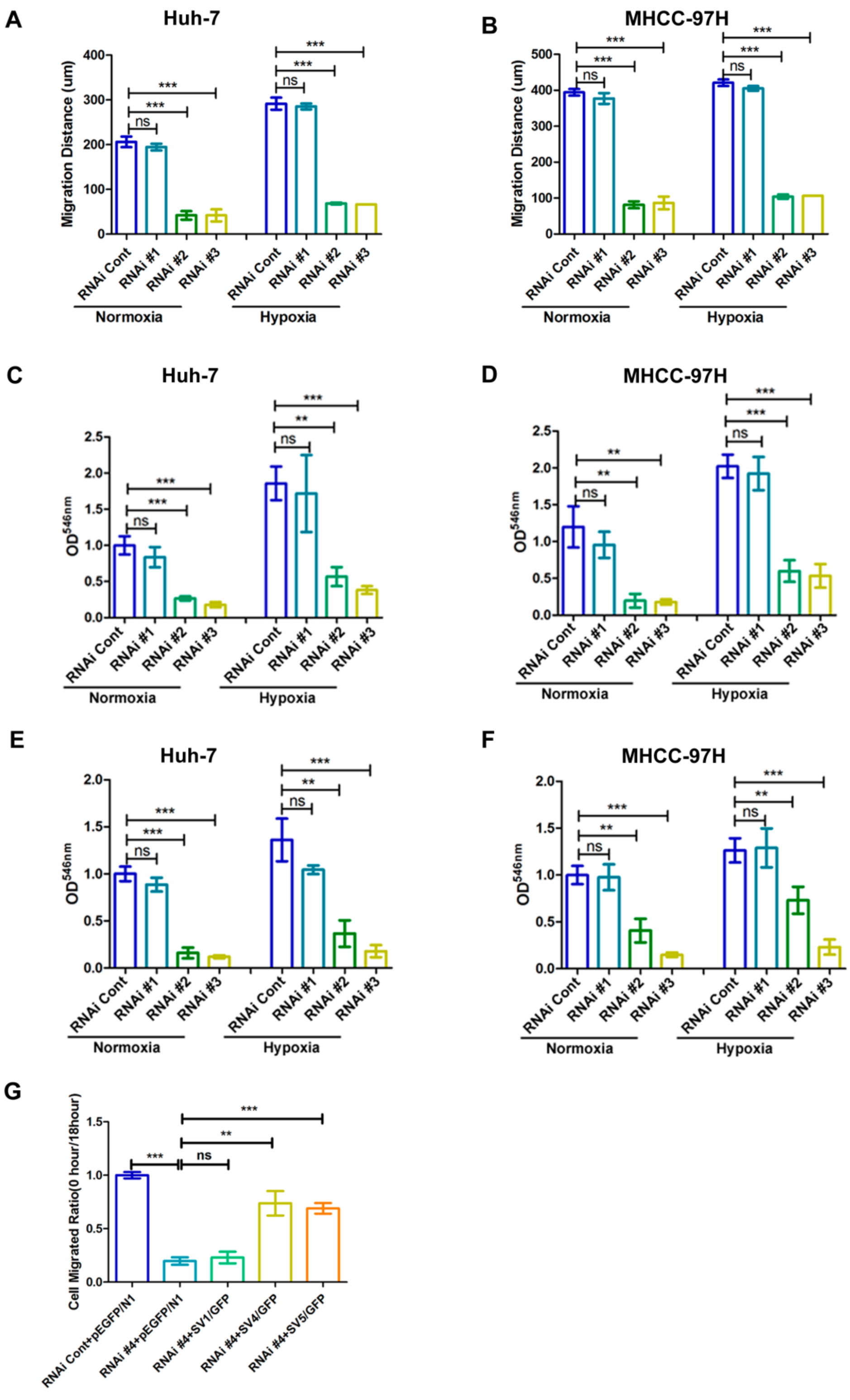

Supplementary Figure S2. Supervillin promotes HCC migration and invasion during hypoxia. A, B. Huh-7 (A) and MHCC-97H (B) cell mobility was detected by wound healing assay. Cells were transfected with control or supervillin-specific siRNA and incubated under normoxic conditions for 48 h, scratched and exposed to

normoxia, or hypoxia for 18 h, respectively. The closure of the scratch was monitored. **C, D.** Huh-7 (C) and MHCC-97H (D) cell migration were detected by Boyden Chamber Transwell assays. Cells were transfected with control or supervillin-specific siRNA and incubated under normoxic conditions for 48 h, after which they were seeded into Transwell chambers for 18 h under normoxia or hypoxia. The number of migrated cells was monitored. **E, F.** Huh-7 (E) and MHCC-97H (F) cell invasion were detected by Boyden Chamber Transwell assays. Cells were transfected with control or supervillin-specific siRNA and incubated under normoxic conditions for 48 h, seeded into Matrigel-coated Transwell inserts, and incubated under normoxia or hypoxia for 18 h. The number of invaded cells was monitored. **G.** MHCC-97H cells that had been treated with supervillin-specific siRNA (RNAi #4, targeted for the supervillin 3′UTR) were allowed to recover their expression of SV1, SV4, and SV5 before assay for cell migration in Transwell chambers under hypoxia for 18 h. The migrated cells on the upper side (at 0 h) and lower side (18 h) were monitored. All of the treatments in this figure were carried out in triplicate, and the results were displayed as the means ± SD.

Supplementary Figure S3

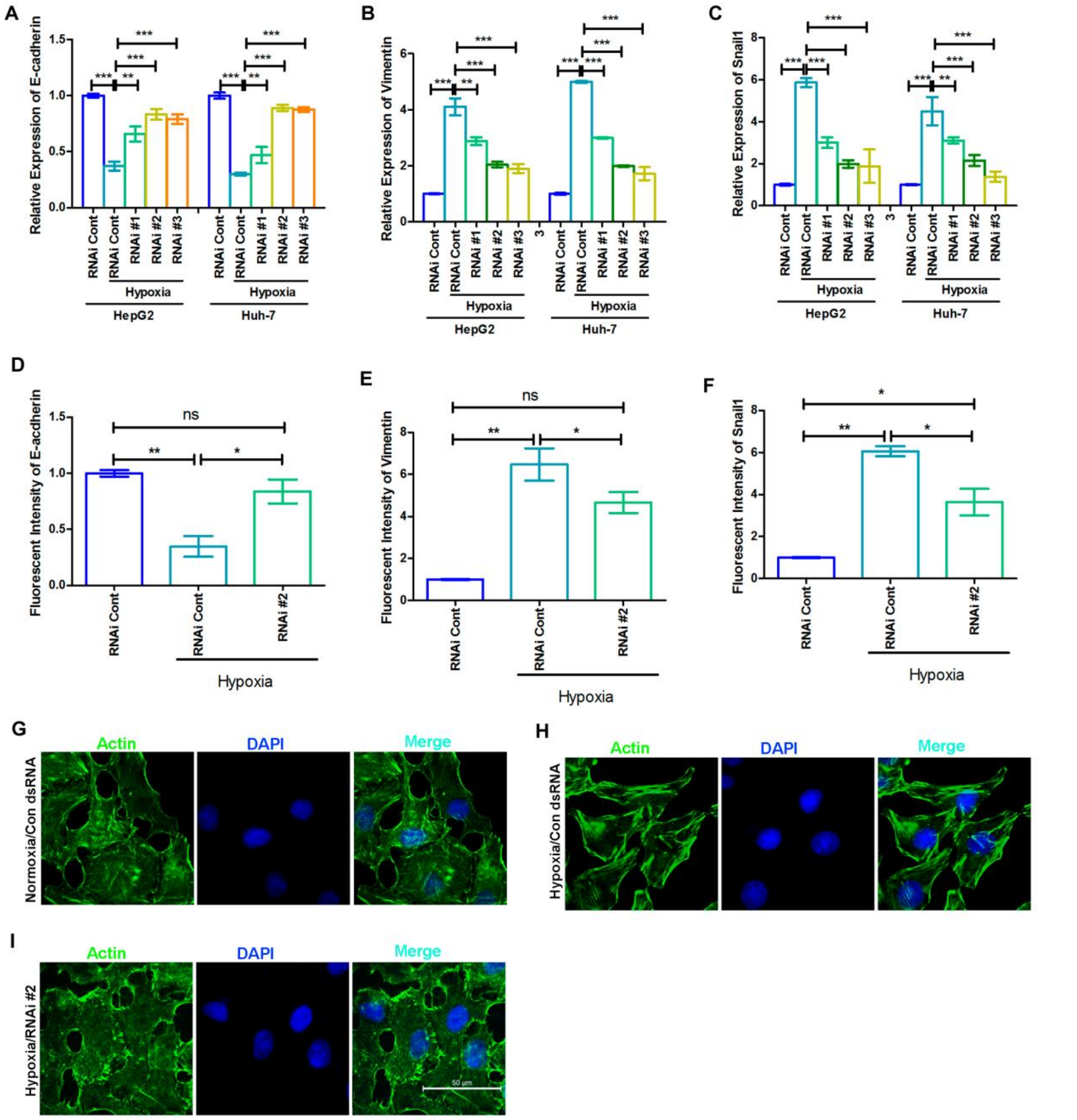

Supplementary Figure S3. Quantification of EMT markers' expression levels regulated by supervillin during hypoxia. A-C. Quantification of immunoblots indicated the changes in E-cadherin, Vimentin, Snail1, and supervillin isoforms in hypoxic conditions after treatment with supervillin-specific siRNA in HepG2 and Huh-7 cells. All of the treatments in this figure were carried out in triplicate, and the results were displayed as the means ± SD. **D-F.** Quantification of immunofluorescence staining indicated the relative fluorescent intensity of E-cadherin, Snail1, and Vimentin under normal and hypoxic conditions in Huh-7 cells treated with control or supervillin-specific siRNA. All of the treatments in this figure were carried out in triplicate, and the results were displayed as the means ± SD. **G-I.** Fluorescence micrographs showing phalloidin-stained F-actin under normal and hypoxic conditions in Huh-7 cells treated with control or supervillin-specific siRNA.

Supplementary Figure S4

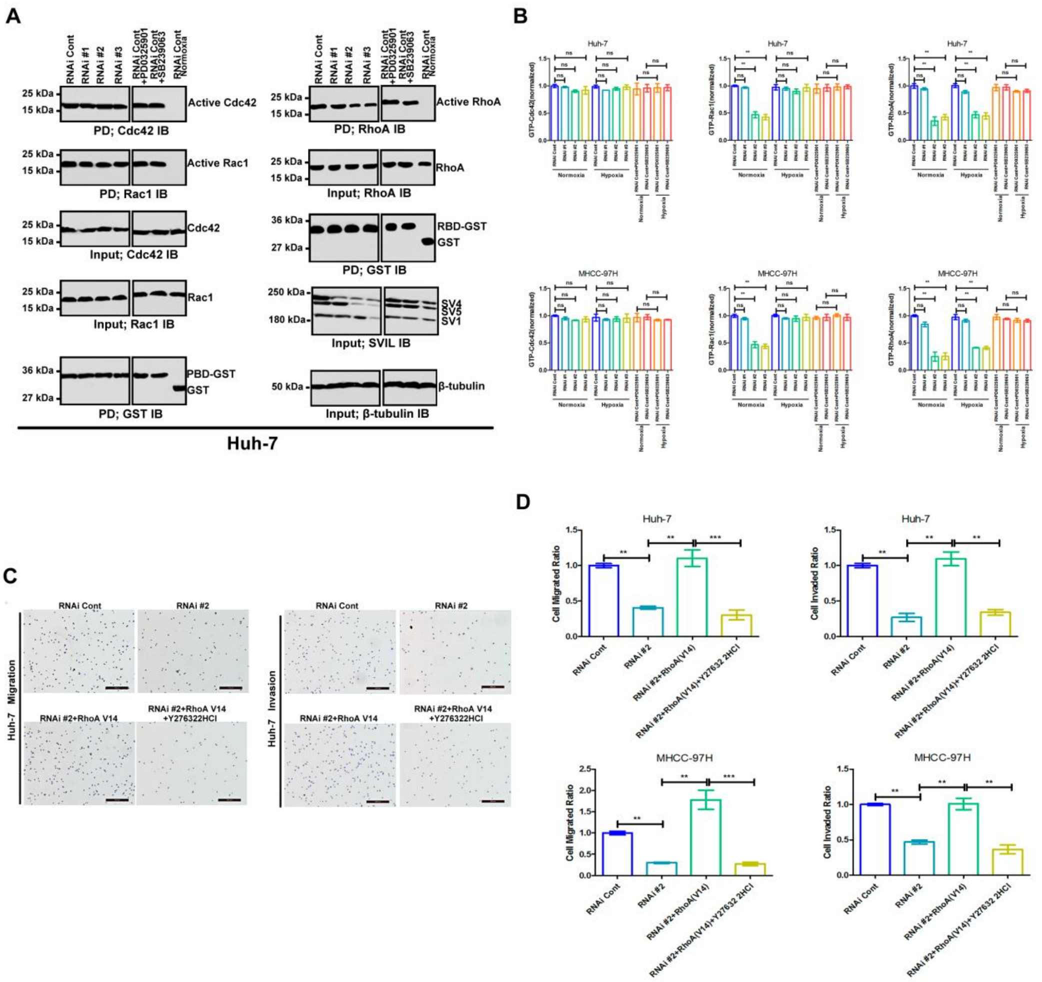

Supplementary Figure S4. Supervillin regulated HCC cell migration and invasion through the RhoA/ROCK pathway during hypoxia. A. Huh-7 cells were transfected with control or supervillin-specific siRNA for 48 h, exposed to hypoxia for 16 h, and lysates were assayed for the relative amounts of GTP-loaded (activated) Rac1, Cdc42, and RhoA. Cells that had been transfected with control RNAi were treated with a MEK inhibitor (PD0325901) or a p38 inhibitor (SB239063) before assaying for GTP-Rac1, Cdc42, and RhoA levels (right). **B.** Quantification of the relative amounts of GTP-loaded (activated) Rac1, Cdc42, and RhoA in Huh-7 and MHCC-97H cells transfected with control or supervillin-specific siRNA for 48 h, exposed to normoxia (left), or hypoxia (middle) for 16 h. All of the treatments in this figure were carried out in triplicate, and the results were displayed as means ± SD. **C.** Huh-7 cells that had been transfected with control or supervillin-specific RNAi were treated with the ROCK inhibitor Y27632 2HCl, incubated for 18 h in 1% O_2, and tested for migratory activity in Boyden Chamber Transwell assays.**,** **D.** Quantification of the number of migrated or invaded Huh-7 and MHCC-97H cells transfected with control or supervillin-specific siRNA, treated with the ROCK inhibitor Y27632 2HCl, and incubated for 18 h in 1% O_2. All of the treatments in this figure were carried out in triplicate, and the results were displayed as means ± SD.

Supplementary Figure S5

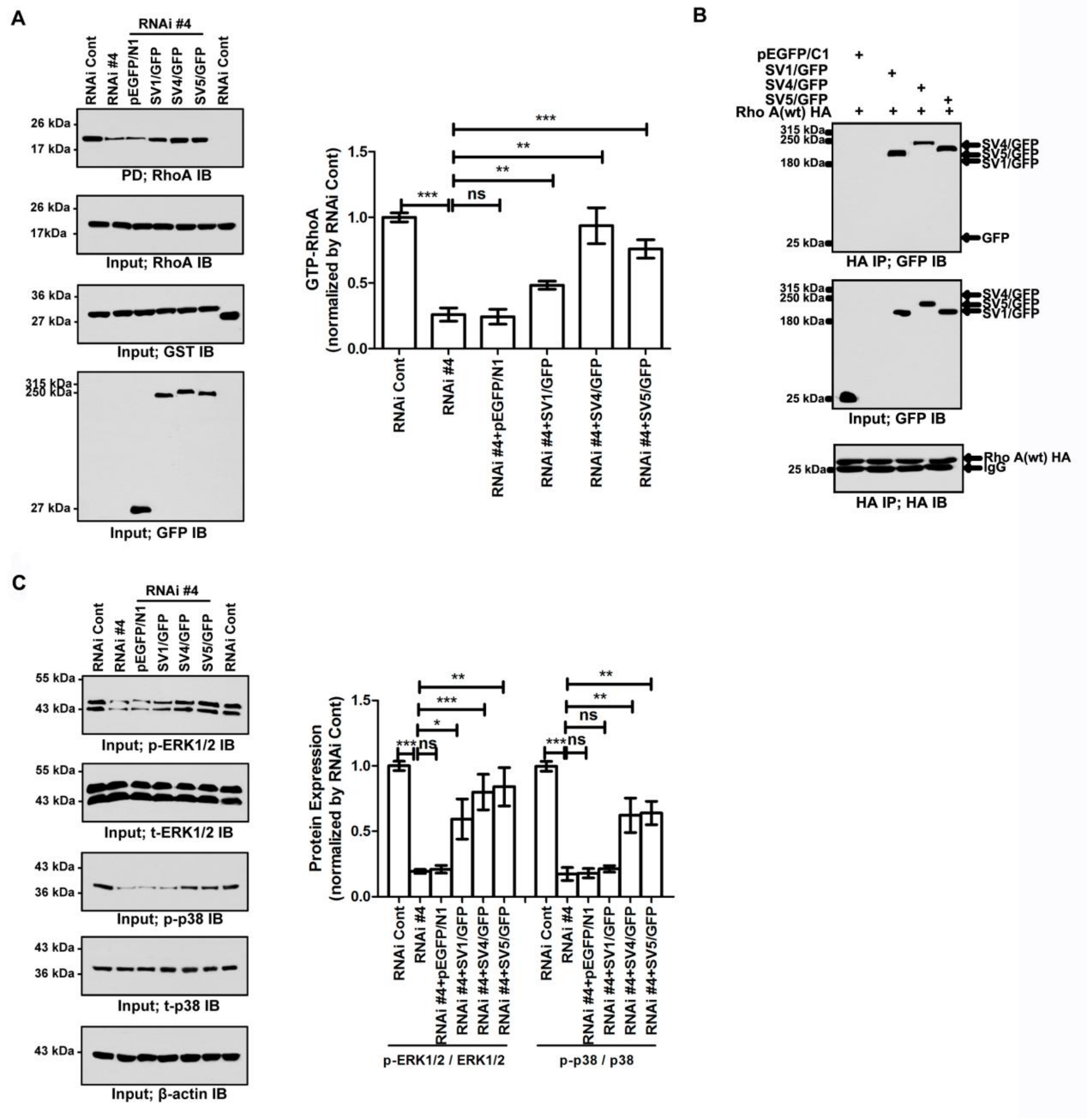

Supplementary Figure S5. Supervillin regulates RhoA activation and ERK/p38 transduction during hypoxia. A, C. MHCC-97H cells that had been treated with control or supervillin-specific siRNA (RNAi #4, which targets the supervillin 3′UTR) were allowed to recover their expression of SV1, SV4, and SV5 and exposed to hypoxia for 18 h. The relative amounts of RhoA-GTP (A), and phosphorylated and total p38 and ERK (C) were determined by immunoblotting. β-actin was used as the loading control. Quantification of GFP-supervillin (SV), p-ERK1/2, and p-p38 levels in hypoxic MHCC-97H cells cotransfected with control or supervillin-specific siRNA #4 and SV1, SV4, or SV5 plasmids. GTP-RhoA, p-ERK1/2, and p-p38 levels were normalized to their respective level in cells transfected with control siRNA. **B.** The interaction between supervillin and RhoA. Cell lysates were prepared from HEK293 cells co-transfected with GFP-tagged SV1, SV4 or SV5 and HA-tagged RhoA. Immunoprecipitation (IP) and immunoblotting (IB) were performed with anti-HA or anti-GFP antibodies.

Supplementary Figure S6

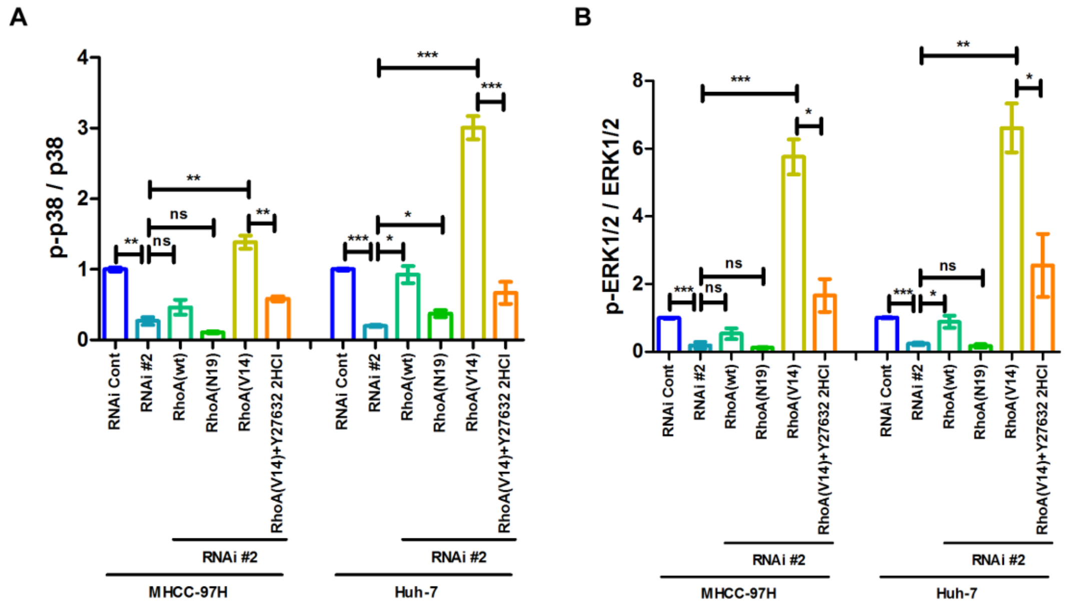

Supplementary Figure S6. Quantification of phosphorylated p38 and ERK levels regulated by supervillin-RhoA/ROCK signaling during hypoxia. Quantification of GFP-SV, p-ERK1/2, and p-p38 levels during hypoxia in MHCC-97H and Huh-7 cells co-transfected with control or supervillin-specific siRNA and RhoA(WT), RhoA(V14), or RhoA(N19) plasmids for 48 h, and then treated with PBS or the ROCK inhibitor Y27632 2HCl (10 μM) for 16 h. Data represent the mean of at least three independent experiments ± SD in Huh-7 and MHCC-97H cells. p-ERK1/2 and p-p38 level was normalized to their respective levels in cells transfected with control siRNA.

Supplementary Figure S7

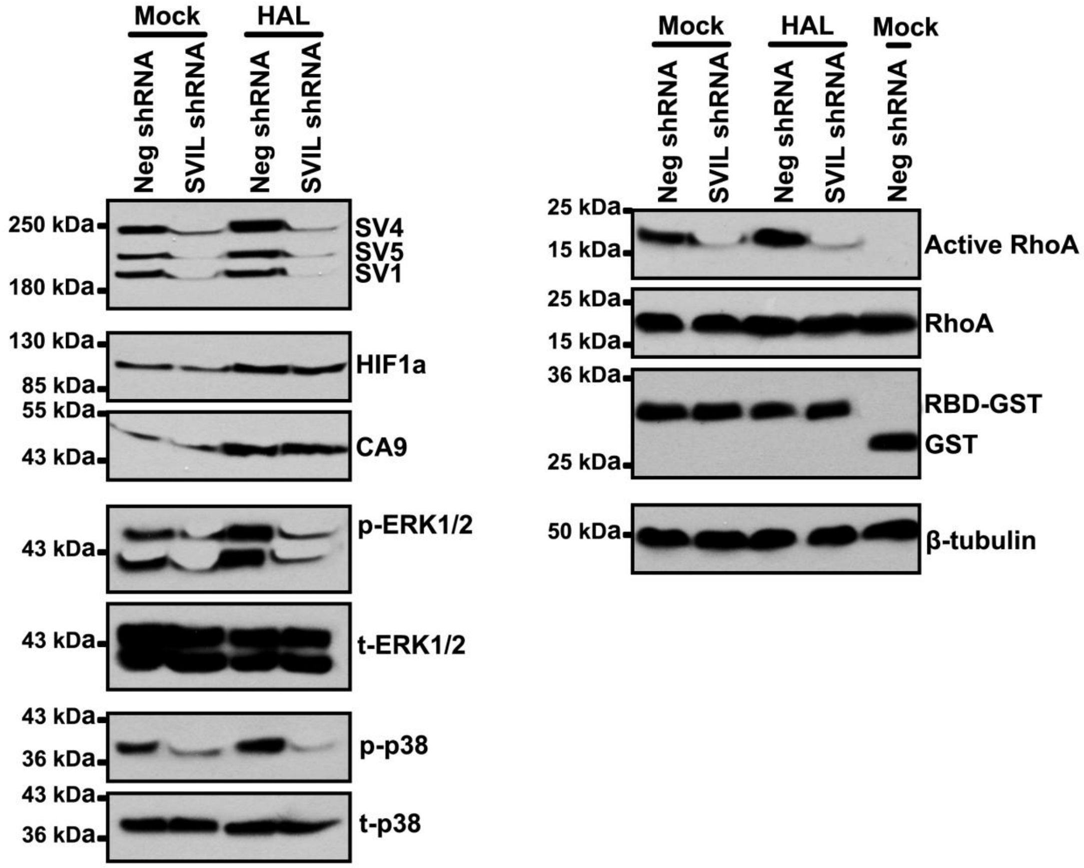

Supplementary Figure S7. Supervillin-RhoA/ROCK-ERK/p38 signaling pathway in HCC metastasis under hypoxia *in vivo*. The tumor form supervillin-knockdown clone or a clone treated with a control shRNA was dissected, lysed, and assayed for the relative amounts of GTP-loaded (activated) RhoA. And the level of supervillin, and the changes in phosphorylated p38, ERK, and JNK were also detected by western blot. β-tubulin was used as the loading control.

Supplementary Figure S8

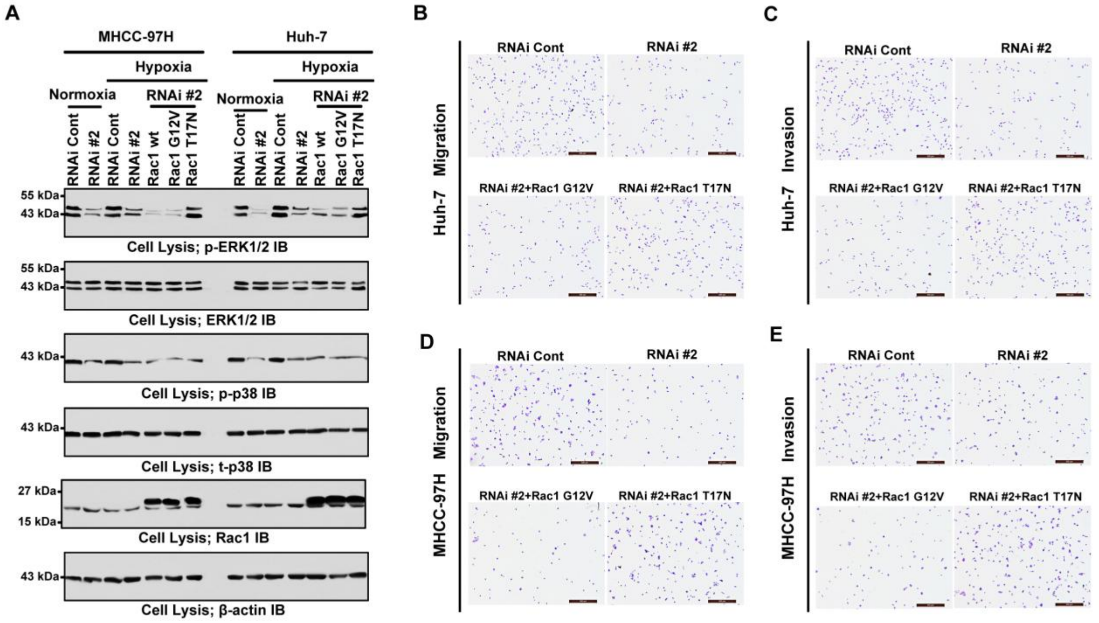

Supplementary Figure S8. Supervillin promotes Rac1 activity and Rac-mediated cell metastasis in normoxia and hypoxia. A. MHCC-97H and Huh-7 cells that had been co-transfected with control or supervillin-specific siRNA and HA-tagged Rac1(WT), Rac1(G12), or Rac1(T17) plasmids were exposed to hypoxia for 18 h. The changes in phosphorylated p38 and ERK were determined by immunoblotting. β-actin was used as the loading control. **B, D.** Huh-7 (B) and MHCC-97H (D) cells that had been co-transfected with control or supervillin-specific siRNA and Rac1(G12) or Rac1(T17) were exposed to hypoxia for 18 h, and then cell migration was detected in Boyden Chamber Transwell assays. **C, E.** Huh-7 (C) and MHCC-97H (E) cells that had been co-transfected with control or supervillin-specific siRNA and constitutively active Rac1(G12) or dominant-negative Rac1(T17) were exposed to hypoxia for 18 h, and then cell invasion was detected using Boyden Chamber Transwell assays.

Supplementary Figure S9

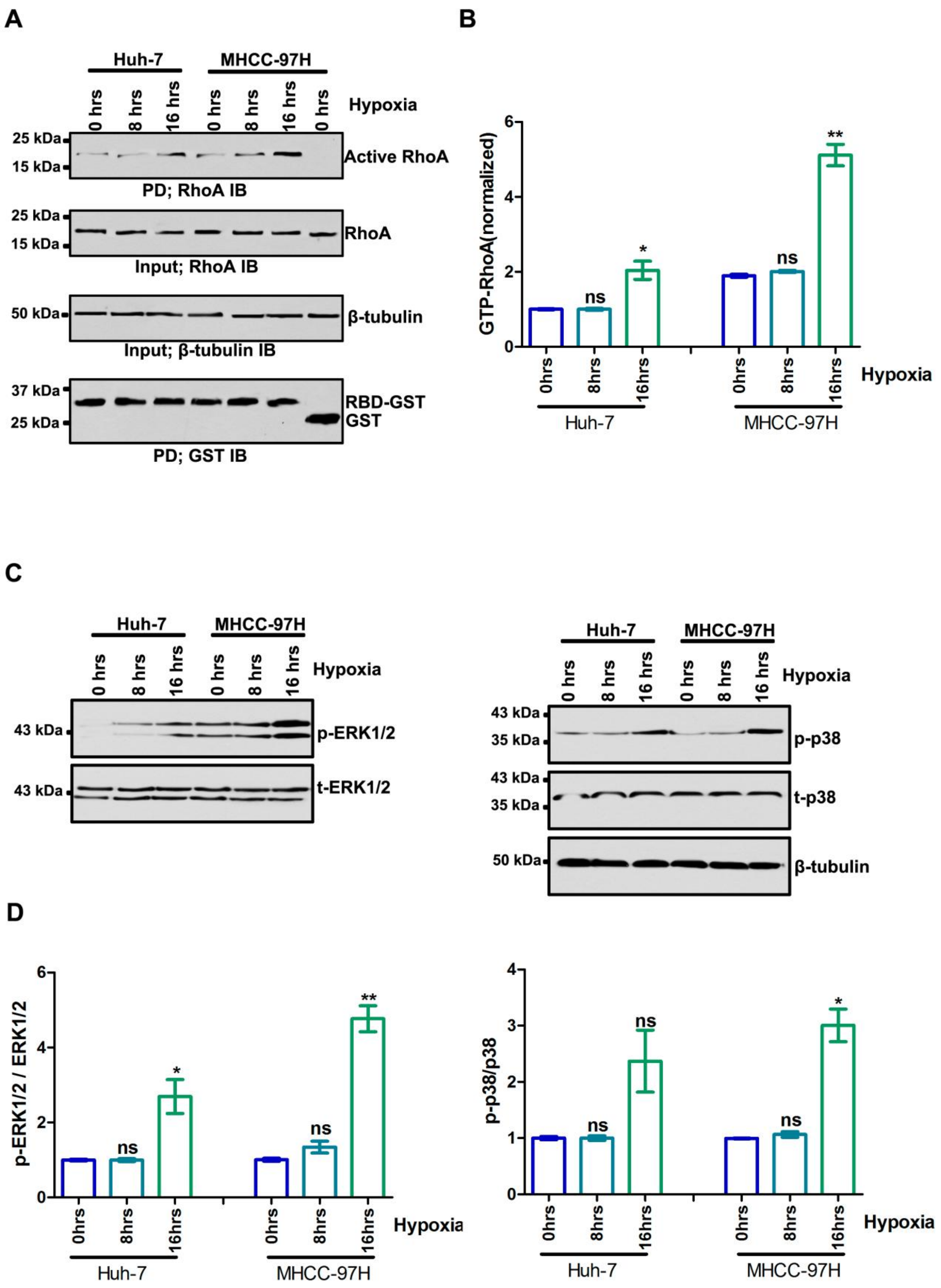

Supplementary Figure S9. RhoA activation and MAPK signaling vary in cell types and environments. A. Huh-7 and MHCC-97H cells were exposed to hypoxia for 16 h, and then lysates were assayed for the relative amounts of GTP-loaded (activated) RhoA. **B.** Quantification of the relative amounts of GTP-loaded (activated)

RhoA in Huh-7 and MHCC-97H cells. Each active RhoA level was normalized to the level in normoxic cells. **C.** Huh-7 and MHCC-97H cells were exposed to hypoxia for 16 h, and then the changes in phosphorylated p38, and ERK were detected by western blot. β-tubulin was used as the loading control. **D.** Quantification of the p-ERK1/2 or p-p38 level in hypoxic Huh-7 and MHCC-97H cells. Each p-ERK1/2 or p-p38 level was normalized to the level in normoxic cells.

Supplementary Figure S10

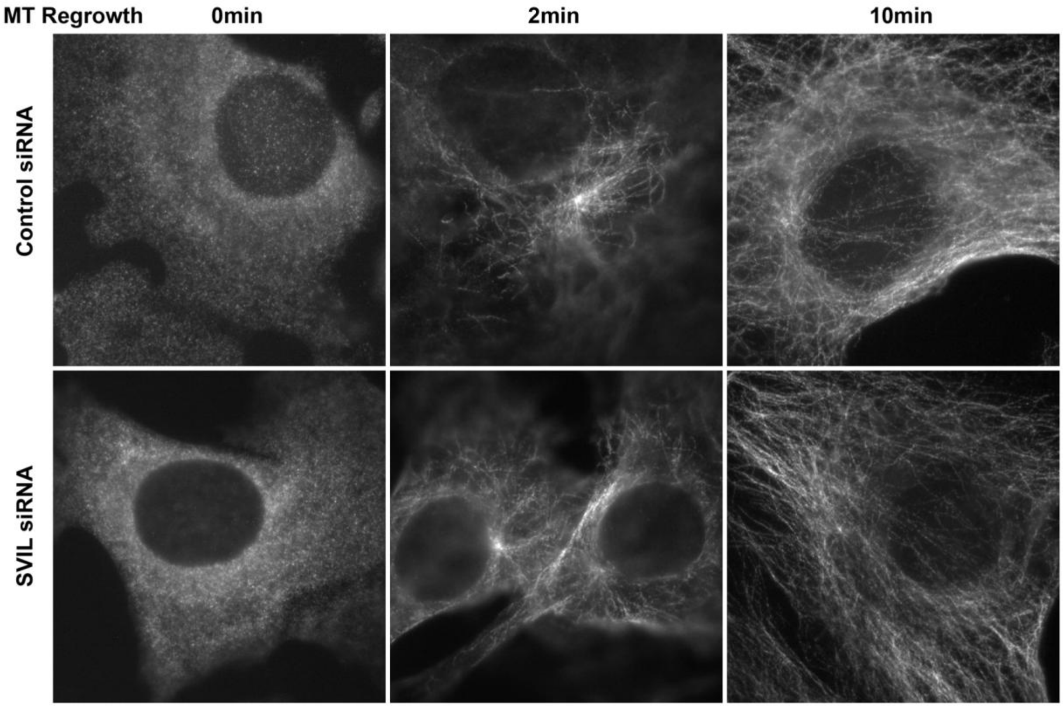

Supplementary Figure S10. There was not obvious effect on microtubule dynamics upon supervillin knockdown. HCC cells were fixed at 2 and 10 min time-points after nocodazole washout then stained for MTs (α-tubulin), and visualized under a fluorescence microscope.

Supplementary Figure S11

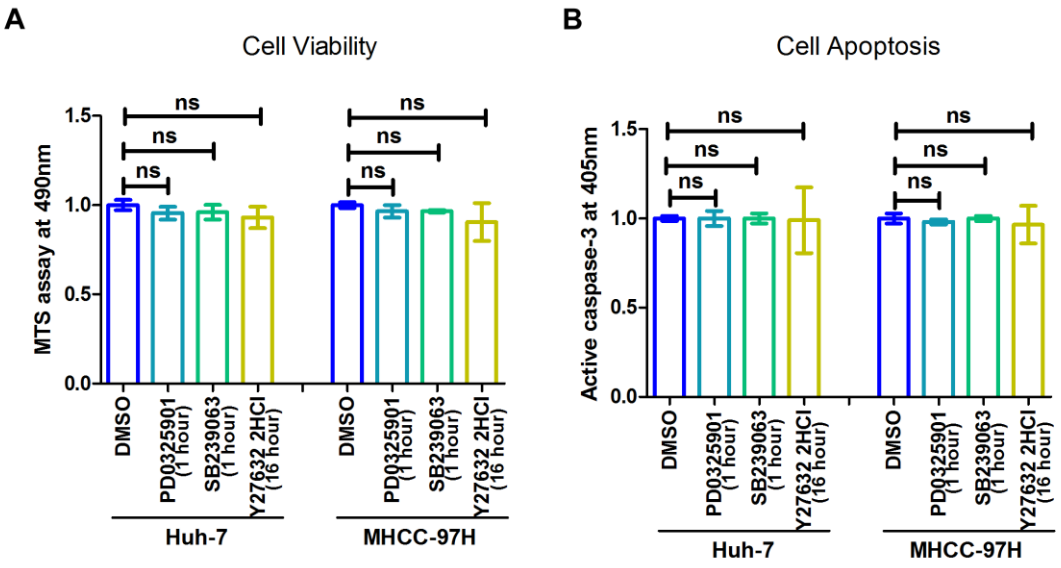

Supplementary Figure S11. The inhibitors used in this study did not affect cell apoptosis and viability. A. The cellular viability of Huh-7 and MHCC-97H cells with MEK inhibitor (PD0325901; 10 µM, 1 h), p38 inhibitor (SB239063; 10 µM, 1 h), or ROCK inhibitor (Y27632 2HCl; 10 µM, 16 h) treatment was determined by MTS (3-(4,5-diethylthiazol-2-yl)-5-(3-carboxymethoxyphenyl)-2-(4-sulfophenyl)-2H-etraz olium,inner salt) assay. All of the treatments in this figure were carried out in triplicate, and the results were displayed as means ± SD. **B.** The level of active caspase-3 in Huh-7 and MHCC-97H cells with the MEK inhibitor (PD0325901; 10 µM, 1 h), p38 inhibitor (SB239063; 10 µM, 1 h), or ROCK inhibitor (Y27632 2HCl; 10 µM, 16 h) treatment was determined. All of the treatments in this figure were carried out in triplicate, and the results were displayed as means ± SD.

Supplementary Table 1. Stealth siRNA used in the study.

Stealth siRNA targeted for human Supervillin		
**No.**	**Location**	**Sequence**
RNAi #1	Exon 4	CUCACUUUGAAUGUAGAGAACCAUC
RNAi #2	Exon 5	UUCUGCUGAAGUUAUAGGUUGGGUU
RNAi #3	Exon 10	AGCAUAUUUAGAUUCCUUAUGGCUG
RNAi #4	3’UTR	UAUUAAGGUAGAAAGGUUGAUUCGC
Note: All Exon located in Supervillin splicing isoform 4(SV4).		

Stealth siRNA targeted for human HIF1α	
**No.**	**Sequence**
RNAi #1	ACUUAAGAAGGAACCUGAUGCUUUA
RNAi #2	CAUCACUUUCUUGGAAACGUGUAAA
